# Effectiveness of conservative management versus laparoscopic cholecystectomy in the prevention of recurrent symptoms and complications in adults with uncomplicated symptomatic gallstone disease (C-GALL trial): pragmatic, multicentre randomised controlled trial

**DOI:** 10.1136/bmj-2023-075383

**Published:** 2023-12-06

**Authors:** Irfan Ahmed, Jemma Hudson, Karen Innes, Rodolfo Hernández, Katie Gillies, Rebecca Bruce, Victoria Bell, Alison Avenell, Jane Blazeby, Miriam Brazzelli, Seonaidh Cotton, Bernard Croal, Mark Forrest, Graeme MacLennan, Peter Murchie, Samantha Wileman, Craig Ramsay, C-GALL Study Group, Abigail Patrick, Aleasha Udal, Alexandra Smith, Alison Lewis, Alison Moss, Ami Laidlaw, Ami Mackay, Ami Wilkinson, Amy Nicol, Andrea Kay, Andrew Beamish, Andrew McDarby, Andrew Robertson, Andrew Torrance, Angela Day, Angela Doyle, Anissa Benchiheub, Antonio Foliaki, Arlo Whitehouse, Ashraf Rasheed, Aya Musbahi, Beena David, Brian Davidson, Bridget Campbell, Bussa Gopinath, Carina Galpin, Carol Dalton, Caroline Young, Cerian Williams, Charmaine Shovelton, Christine Eastgate, Christopher Halloran, Claire McNeill, Claire Price, Corinne Pawley, Dave Gribble, David Chadwick, David O’Reilly, David Stell, Debbie Wilson, Debra Campion, Deena Harji, Diana Nayman, Dipankar Chattopadhyay, Edward Lopez, Elaine Wall, Elizabeth Cornes, Elizabeth Williams, Esther Zebracki, Evelyn Baker, Ewen Griffiths, Fady Yanni, Faye Moore, Frances Venn, Frances Walsh, Francesca Westwell, Gabriele Marangoni, Gail Williams, Geert Koffeman, Georgia Mallison, Gillian Rice, Godwin Dennison, Grace Okoro, Hamish Noble, Heeam Nassa, Helder Filipe, Indy Atwal, Ish Ahmed, James Catton, James Milburn, Jamie Young, Jawad Ahmad, Jemma Tuffney, Jenny Porter, Jess Perry, Joanna Allison, Jody Carvell, John Saunders, John Spearman, Jolene Witherspoon, Joy Dawson, Judith Rozentals, Julie Colley, Julie Haydock, Julie Markley, Kanapathi Rajaratnam, Karen Spicer, Karim Sillah, Karlygash Ausel, Kate Beesley, Kate Marks, Kathryn Webb, Katrina Butcher, Keith Boath, Keith Roberts, Kurinchi Gurusamy, Kyaw Toe, Laura Beveridge, Laura Jones, Laura Smith, Laura Stephen, Leo Brown, Liam Preece, Libby Graham, Linda Howard, Lindianne Aitken, Liz Baker, Lorna Fleming-Bird, Lorna Van Lierop, Louise Duncan, Lucy Knibbs, Manijeh Ghods, Maria Laye, Maria O’Leary, Marianne Hollyman, Markos Daskalakis, Martin Grigg, Maryam Hussain, Matthew Mason, Mazuin Abutalib, Mel Penacerrada, Michael Nutt, Milind Rao, Mokhtar Eltair, Momin Malik, Murugappan Shanmugam, Muwaffaq Telfah, Nancy Hawkings, Natalie Kenny, Natasha Wilmshurst, Nathan Curtis, Neil Welch, Nilanjana Tewari, Olga Tucker, Paul Glen, Paul Marriott, Paul Super, Penny Parsons, Peter Driscoll, Peter Mekhail, Raj Nijjar, Rajendirin Ashwin, Rajnish Mankotia, Ravi Marudanayagam, Ravinder Vohra, Rebecca Goodchild, Retno Walandari, Rezwana Chowdhury, Rhian Warman, Richard Welbourn, Rishi Singhal, Robert Sutcliffe, Safia Begum, Sally Abbott, Sam Dutta, Samantha Stafford, Samia Hussain, Sandapa Punchihewa, Sandra Mann, Sarah Brown, Sarah Clark, Sharon Garner, Shirley Pyke, Siddek Isreb, Simon Gibson, Simon Parsons, Sonia Puig-Compano, Sophie Mason, Stef Hobson, Steven Henderson, Sue Pick, Susan Dale, Suvi Virupaksha, Tabinda Kharodia, Talvinder Gill, Tanvir Hossain, Tracy Brear, Tracy Scott, Veronica Campbell, Vasileios Charalampakis, Viktoria Cripps, VJ Shabangu, Yogesh Kumar, Yvonne Gleeson, Zaher Toumi

**Affiliations:** 1 Department of Surgery, NHS Grampian, Aberdeen, UK; 2 Health Services Research Unit, University of Aberdeen, Aberdeen, UK; 3 Health Economics Research Unit, University of Aberdeen, Aberdeen, UK; 4 Centre for Surgical Research, NIHR Bristol and Western Biomedical Research Centre, University of Bristol, Bristol, UK; 5 Clinical Biochemistry, NHS Grampian, Aberdeen, UK; 6 The Centre for Healthcare Randomised Trials,Health Services Research Unit, University of Aberdeen, Aberdeen, UK; 7 Academic Primary Care, Institute of Applied Health Sciences, University of Aberdeen, Aberdeen, UK

## Abstract

**Objective:**

To assess the clinical and cost effectiveness of conservative management compared with laparoscopic cholecystectomy for the prevention of symptoms and complications in adults with uncomplicated symptomatic gallstone disease.

**Design:**

Parallel group, pragmatic randomised, superiority trial.

**Setting:**

20 secondary care centres in the UK.

**Participants:**

434 adults (>18 years) with uncomplicated symptomatic gallstone disease referred to secondary care, assessed for eligibility between August 2016 and November 2019, and randomly assigned (1:1) to receive conservative management or laparoscopic cholecystectomy.

**Interventions:**

Conservative management or surgical removal of the gallbladder.

**Main outcome measures:**

The primary patient outcome was quality of life, measured by area under the curve, over 18 months using the short form 36 (SF-36) bodily pain domain, with higher scores (range 0-100) indicating better quality of life. Other outcomes included costs to the NHS, quality adjusted life years (QALYs), and incremental cost effectiveness ratio.

**Results:**

Of 2667 patients assessed for eligibility, 434 were randomised: 217 to the conservative management group and 217 to the laparoscopic cholecystectomy group. By 18 months, 54 (25%) participants in the conservative management arm and 146 (67%) in the cholecystectomy arm had received surgery. The mean SF-36 norm based bodily pain score was 49.4 (standard deviation 11.7) in the conservative management arm and 50.4 (11.6) in the cholecystectomy arm. The SF-36 bodily pain area under the curve up to 18 months did not differ (mean difference 0.0, 95% confidence interval −1.7 to 1.7; P=1.00). Conservative management was less costly (mean difference −£1033, (−$1334; −€1205), 95% credible interval −£1413 to −£632) and QALYs did not differ (mean difference −0.019, 95% credible interval −0.06 to 0.02).

**Conclusions:**

In the short term (≤18 months), laparoscopic surgery is no more effective than conservative management for adults with uncomplicated symptomatic gallstone disease, and as such conservative management should be considered as an alternative to surgery. From an NHS perspective, conservative management may be cost effective for uncomplicated symptomatic gallstone disease. As costs, complications, and benefits will continue to be incurred in both groups beyond 18 months, future research should focus on longer term follow-up to establish effectiveness and lifetime cost effectiveness and to identify the cohort of patients who should be routinely offered surgery.

**Trial registration:**

ISRCTN registry ISRCTN55215960.

## Introduction

Gallstone disease (cholelithiasis) is one of the most common gastrointestinal disorders worldwide, with clinical surveys suggesting prevalence rates of 6% to 25% and a tendency to increase with age.^1^ ^2^ ^3^ ^4^ ^5^ ^6^ ^7^ ^8^ ^9^ ^10^ Gallstones are more common in women.^6^ A clinical ultrasound survey in the UK reported prevalence rates of 12% and 22% among men and women older than 60 years, respectively.^9^

In the UK and North America, the number of surgical procedures for gallstone disease increased steadily between the 1950s and 1990s, reflecting the rise in prevalence and prompt identification of gallstone disease and the use of cholecystectomy as the treatment of choice.^6^ Rates of surgical procedures stabilised in these countries towards the end of the 20th century.^5^ In England, about 61 000 episodes cost the NHS >£200m in 2018/19.^11^

The natural course of gallstones is benign; most people remain asymptomatic and show a relatively low progression to symptomatic disease.^12^ A systematic review published in 2007 reported the range for disease progression as 10% to 25% in studies that followed-up patients after initial diagnosis (≤15 years of follow-up).^13^ The annual risk of developing symptoms has been estimated at 2-4%.^12^

Most people with symptomatic uncomplicated gallstone disease do not develop complications; reported annual rates of developing complications (eg, acute cholecystitis, acute pancreatitis, acute cholangitis obstructive jaundice) have been as low as 1-3%.^14^ ^15^ ^16^ The Italian Group for the Epidemiology and Prevention of Cholelithiasis study reported an annual incidence for complications of 0.7% in patients with symptoms.^17^

Mortality from gallstone disease is rare—typically less than 1% from gallstone related causes.^12^ ^17^ ^18^

From a patient perspective, the defining symptom of gallstone disease is severe and lasting (ie, >30 minutes) abdominal pain.^19^ ^20^ General abdominal symptoms commonly intensify over a period and become regular pain attacks (biliary colic) that may require medical attention.

A recent large prospective study in the UK (n=8909 participants) showed that 10.8% of people experienced complications 30 days after surgery.^21^ Furthermore, as much as 40% of patients may continue to experience pain and abdominal symptoms after surgery.^22^ In particular, persistent pain similar to that experienced preoperatively has been reported in about 20% of people after cholecystectomy,^23^ ^24^ and new pain has been reported in up to 14% of people^25^ Based on the 61 000 episodes reported for 2018/19, 6600 people would have experienced complications from surgery and 24 400 would continue to experience pain, with a substantial impact on NHS resources and costs.^11^

Post-cholecystectomy syndrome is an umbrella term widely used to describe the range of symptoms patients might experience after surgery.^26^ The term persistent post-cholecystectomy symptoms has been suggested as a more accurate description of these symptoms,^27^ which include biliary and non-biliary abdominal pain, dyspepsia, heartburn, nausea, vomiting, and jaundice. Up to 40% of people may experience persistent pain and discomfort, usually described as post-cholecystectomy symptoms.^22^ ^25^ Persistent diarrhoea or constipation are often reported after cholecystectomy, and flatulence may arise as a new symptom.^25^ ^28^ ^29^

The C-GALL (laparoscopic cholecystectomy versus observation/conservative management for preventing recurrent symptoms and complications in adults with uncomplicated symptomatic gallstones) trial examined the clinical and cost effectiveness of conservative management compared with laparoscopic cholecystectomy to prevent recurrent symptoms and complications in adults with uncomplicated symptomatic gallstone disease.

## Methods

### Study design and participants

C-GALL was a pragmatic, multicentre, parallel group, patient randomised, superiority trial to test if the strategy of standard, laparoscopic cholecystectomy is more effective and cost effective than conservative management. The trial protocol was published in 2021.^30^

We recruited patients from 20 secondary care sites in the UK. Potential participants were adults aged >18 years with confirmed, symptomatic uncomplicated gallstone disease (ie, biliary colic or acute cholecystitis) who were electively referred to secondary care and considered suitable for cholecystectomy. Clinical diagnosis of gallstone disease was confirmed by appropriate imaging. Patients who were unable to consent, medically unfit for surgery, pregnant, or had had previous open major upper abdominal surgery were not eligible for the trial. Also excluded were patients with gallstones in the common bile duct, evidence of previous choledocholithiasis, a history of acute pancreatitis, evidence of obstructive jaundice, evidence of empyema of the gallbladder with sepsis, suspicion of gallbladder cancer, perforated gallbladder (recent or old perforation detected on imaging), or haemolytic disease.

### Randomisation and masking

Participants were randomly assigned (1:1) to receive either laparoscopic cholecystectomy or conservative management using the remote, computer based randomisation application at the Centre for Healthcare Randomised Trials. The minimisation algorithm used recruitment site, sex (male, female), and age (<35, 35-64, ≥65 years). A random element (20% chance) was incorporated into the minimisation algorithm. Participants, investigators, and the trial statistician were not masked to treatment allocation.

### Procedures

*Laparoscopic cholecystectomy*—Surgical management, performed under general anaesthesia, remains the current standard procedure for symptomatic gallstone disease. Occasionally it may be necessary during the procedure to convert to open surgery because of a complication or difficulty in progressing safely. Moreover, an alternative procedure may be performed if difficulty is anticipated in removing the gallbladder safely (eg, drainage of the gallbladder, subtotal cholecystectomy).

*Conservative management*—Conservative management for gallstone disease can involve observation, and the prescription of analgesics when needed to relieve biliary pain, and it is largely based in primary care in the community. When required, typical treatment for pain includes paracetamol (acetaminophen), non-steroidal anti-inflammatory drugs, narcotic analgesics, such as opiates, and antispasmodics (eg, buscopan), together with generic advice on a healthy lifestyle. 

Participants who were randomised to the conservative management group were also given a patient information leaflet about medical management, which included steps to take if symptoms recurred or flared up, and standard NHS advice on a healthy diet for gallstone disease.

### Outcomes

Measurements were taken from participants’ questionnaires at baseline and at 3, 9, 12, and 18 months. The primary outcome was the area under the curve up to 18 months post-randomisation using the short form, SF-36, bodily pain domain (norm based score transformed to align with a general population with a mean of 50 and a standard deviation of 10). Area under the curve was chosen to incorporate the total quality of life of participants throughout the trial. The patient reported secondary outcomes were area under the curve up to 24 months post-randomisation for the SF-36 bodily pain domain, condition specific questionnaire, SF-36 domains (excluding bodily pain), need for further treatment, and persistent symptoms (consisting of two sections (pain and dyspepsia) of the condition specific questionnaire) at 18 months and 24 months after randomisation. The clinical secondary outcome was complications, defined as any complication before, during, or after surgery at 18 months and 24 months after randomisation. Adverse events, serious adverse events, and death were also recorded. Economic outcomes included UK NHS resource use and costs, QALYs obtained with the responses to the SF-36 instrument,^31^ and incremental cost effectiveness ratio measured as the difference in mean cost divided by the difference in mean QALYs between study groups.

Data on NHS hospital inpatient resource use were obtained through hospital case report forms. Data on primary care contacts, secondary outpatient care, and drugs for symptomatic gallstone disease or post-surgery were acquired using participants’ questionnaires at 3, 9, 12, 18, and 24 months post-randomisation. Data on resource use were combined with data on national unit costs for the financial year 2019/20^11^ to obtain total cost per participant up to 24 months post-randomisation. We used participants’ responses to the SF-36 questionnaire at baseline and at 3, 9, 12, 18, and 24 months post-randomisation to estimate SF-6D (short form 6 dimensions) utilities^32^ to calculate total QALYs up to 24 months post-randomisation for each participant. We assumed a linear change in health state utility between data collection time points. Further details of the economic evaluation analysis, including a modelling extrapolation beyond trial follow-up, are reported elsewhere.

### Statistical analysis

A sample size of 194 in each group was needed to detect a mean difference in area under the curve of 0.33 standard deviations derived from the SF-36 bodily pain domain with 90% power and a 5% (two sided α) significance level. As observed in other clinical studies, a difference of 0.33 standard deviations in generic health status is considered clinically relevant in terms of treatment effect size in the small to medium ranges. To allow for 10% of participants with data completely missing, with no area under the curve calculable, we needed 430 participants.

The analysis followed a prespecified statistical analysis plan (see supplementary appendix 1). Analyses were based on the intention-to-treat principle, with participants analysed as randomised irrespective of crossover. We analysed the primary outcome using a mixed effects regression model, with fixed effects for the minimisation covariates and a random effect for the centre. The area under the curve for each participant was generated by the trapezium rule for those with at least one time point up to 30 months post-randomisation. We imputed missing data at 18 months with multiple imputation using Rubin’s rule under a missing at random assumption. A sensitivity analysis was performed including all participants who had at least one time point up to 18 months, with multiple imputation being used for missing data at 18 months. A complete case analysis of the primary outcome was also performed for participants with a score at 18 months, and those without such a score were excluded. The area under the curve is interpreted as bodily pain over 18 months. The results of the condition specific questionnaire, SF-36 (excluding bodily pain), and persistent symptoms were analysed using a repeated measures mixed effects regression model correcting for baseline score, fixed effects for the minimisation covariates, and a random effect for centre. We measured outcomes at 3, 9, 12, and 18 months, and treatment effects were estimated from time-by-treatment interactions at each time point. Missing baseline data were imputed using the centre specific mean of that variable. We analysed complications and need for further treatment using a Poisson model adjusted for minimisation covariates sex (male, female) and age (<35, 35-64, ≥65 years) and including a random effect for the centre using robust error variance.^33^ Planned subgroup analyses explored the potential treatment effect modification of sex (male, female), age (<35, 35-64, ≥65 years), and ethnicity (white versus other, owing to limited data on categories) on the primary outcome using a stricter level of significance (two sided 1% significance level). To assess the effect of compliance on the primary outcome, we used a two stage least squares regression model adjusted for minimisation covariates as fixed effects and adjusted for centre using cluster robust variance. Adherence was defined as participants who received their allocated treatment within 24 months. For the cholecystectomy group, participants who received emergency cholecystectomy were defined as non-adherent. We did not measure adherence with the standard NHS advice on a healthy diet for gallstone disease provided to participants. Continuous variables were summarised using mean (standard deviation), or median and interquartile range, whereas discrete variables were reported as absolute number and percentage in each category. To assess the impact of covid-19, we undertook a sensitivity analysis on the primary outcome for the subset of data pre-covid-19 (data not shown) using the same analysis as described above. Analyses were carried out using Stata statistical software, release 16.

The economic analysis was conducted according to a prespecified and agreed health economics analysis plan (available from the corresponding author on request). Mirroring the statistical analysis, the principles of the intention-to-treat analysis were followed to compare cost and QALYs between groups. Data for the 24 month follow-up were used as these would better reflect costs and consequences relevant to the economic analysis. Reliance on complete case data for cost effectiveness analysis can introduce bias unless the data are completely missing at random. We implemented multiple imputation^34^ as part of the primary economic analysis, using chained equations with predicted mean matching and generating 20 imputed datasets with plausible fitted values assigned for missing cost and utility elements. The imputation model included all the variables in the analysis model (age, sex, treatment group allocation), and auxiliary variables that may help to explain missingness (trial centre, indicator for having surgery, and type of procedure). We used Rubin’s rules to pool estimates across multiple imputation datasets.^35^ General linear regression models adjusted for minimisation factors (centre, age, sex) and baseline SF-6D score were used. Adjusted mean values by treatment allocation, and the incremental difference between the groups were obtained using the methods of recycled predictions.^34^ The incremental cost effectiveness ratio was calculated as the difference in mean costs divided by the difference in mean QALYs for the conservative management group versus the cholecystectomy group. Uncertainty surrounding the joint incremental costs and effects was characterised using non-parametric bootstrapping using 1000 iterations, with the multiple imputation process (κ=5 and 20 simulated datasets) nested within the bootstrapping process.^36^ Based on the bootstrap iterations, we report 95% credible intervals and the probability of the interventions being cost effective at the £20 000 cost effectiveness threshold, following guidance from the National Institute for Health and Care Excellence.^37^

### Patient and public involvement

The two patient and public involvement (PPI) partners (one grant holder/member of the project management group, and one independent member of the trial steering committee) were actively involved in discussions of the study results with the trial steering committee and the trial investigators, and they contributed to preparing the plain English summary. The PPI group (which was initially established as a focus group for the Core Outcome Set^38^ but remained actively involved in the wider C-GALL project) was actively involved in discussions of the study results with the PPI partners and contributed to reviewing the plain English summary. At the conclusion of the study, the PPI partners reflected on their input and made suggestions for future research.

## Results

**Fig 1:**
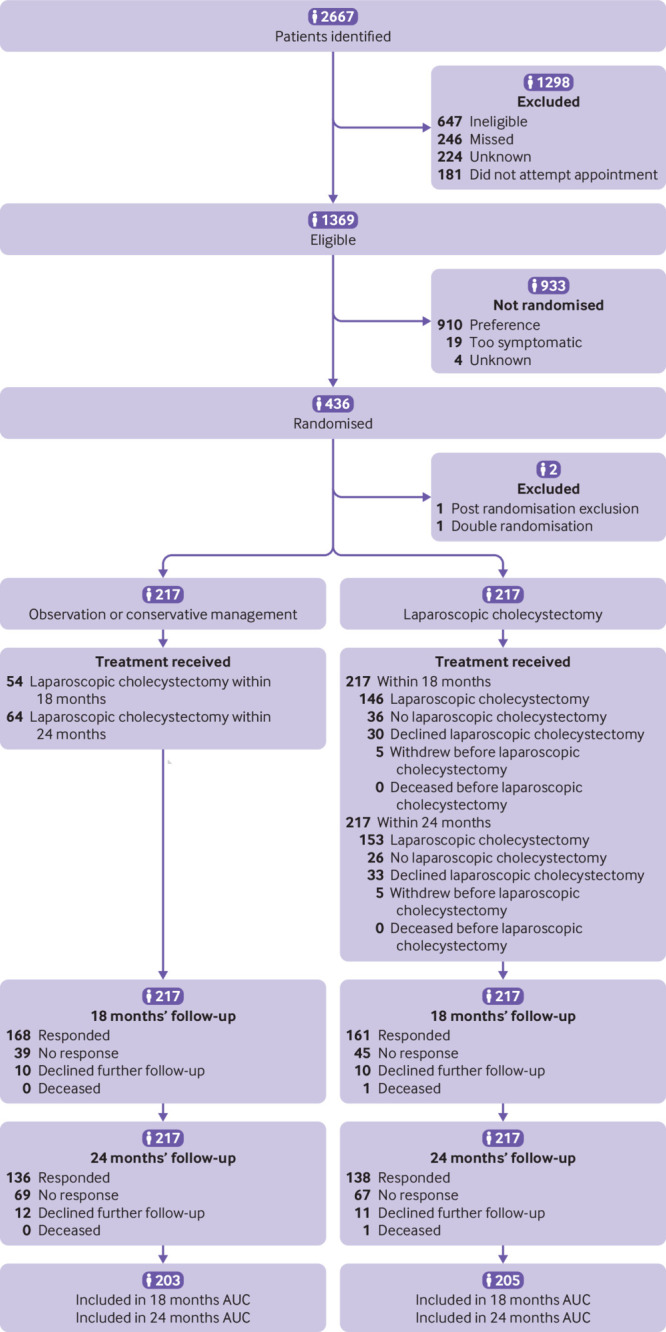
Trial profile (also see supplementary appendix 2, table S1, Reasons for preference). AUC=area under the curve

Overall, 2667 patients in 20 secondary care centres were assessed for eligibility between August 2016 and November 2019. Of these, 436 were randomised to receive either conservative management (n=218) or laparoscopic cholecystectomy (n=218; fig 1). After two post-randomisation exclusions, 434 participants were included; one participant withdrew from the conservative management group owing to a previously unstated preference for laparoscopic cholecystectomy, and one participant was randomised twice to the cholecystectomy group. For the primary outcome analysis, 217 participants were included in the conservative management group and 217 in the cholecystectomy group. The randomised groups were well balanced at baseline (table 1).

**Table 1:** Baseline characteristics. Values are number (percentage) unless stated otherwise

Characteristics	Conservative management (n=217)	Laparoscopic cholecystectomy (n=217)
Mean (SD) age (years); No	50.4 (15.1); 217	50.5 (15.3); 217
Sex:		
Men	46 (21)	47 (22)
Women	171 (78)	170 (78)
Ethnicity:		
White	185 (85)	188 (87)
Mixed/multiple ethnic groups	2 (1)	1 (0.5)
Asian/Asian British	15 (7)	15 (7)
Black/African/Caribbean/black British	7 (3)	5 (2)
Arab	-	2 (1)
Other	7 (3)	6 (3)
Missing	1 (0.5)	-
Mean (SD) BMI; No	32.0 (7.0); 215	31.5 (7.1); 217
Diabetes:		
None	200 (92)	203 (94)
Type 1	-	2 (1)
Type 2	17 (8)	12 (6)
Gallbladder wall*:		
Normal	131 (60)	120 (55)
Thick	27 (12)	30 (14)
Not recorded	59 (27)	67 (31)
Mean (SD) thickness if gallbladder wall thick* (mm); No	5.3 (2.1); 10	5.9 (3.4); 15
Hypertension:		
No	173 (80)	182 (84)
Yes	43 (20)	35 (16)
Missing	1 (0.5)	-
Mean (SD) SF-36 norm based scores; No:		
Bodily pain	44.5 (11.7); 215	43.3 (11.1); 216
Physical functioning	48.2 (10.6); 214	47.3 (10.9); 216
Role physical	47.7 (10.3); 215	46.4 (11.4); 216
General health	45.0 (9.3); 213	43.3 (10.4); 216
Vitality	46.7 (10.0); 213	44.7 (10.9); 216
Social functioning	45.6 (11.7); 213	43.9 (12.5); 216
Role emotional	45.9 (12.4); 215	44.7 (13.3); 216
Mental health	47.7 (10.4); 213	46.1 (11.1); 216
PCS	46.7 (9.3); 213	45.6 (9.7); 216
MCS	46.4 (11.5); 213	44.72 (12.1); 216
Mean (SD) Otago gallstones CSQ†; No	33.2 (19.9); 210	35.4 (20.6); 211
Mean (SD) persistent symptoms score†; No	43.0 (20.9); 213	44.6 (22.8); 215

BMI=body mass index; CSQ=condition specific questionnaire for gallstones; MCS=mental component summary; PCS=physical component summary; SD=standard deviation.

For SF-36 norm based scores, a higher score (range 0-100) indicates better quality of life. For Otago gallstones CSQ, a higher score (range 0-100) indicates higher symptom burden and therefore poorer quality of life.

* Confirmed by transabdominal ultrasonography or another imaging technique.

† Derived from two CSQ domains, pain and dyspepsia.

By 18 months, 54 (25%) participants in the conservative management group and 146 (67%) in the cholecystectomy group had received surgery. The median time to surgery was 8.1 months (interquartile range 4.0-10.6; n=53) in the conservative management group and 4.5 months (2.7-6.9; n=146) in the cholecystectomy group; 46 (85%) and 142 (97%) were elective surgeries, respectively (see supplementary appendix 2, table S2). By 24 months post-randomisation, 64 (29%) participants in the conservative management group and 153 (71%) in the cholecystectomy group had received surgery. Among the 153 participants in the conservative management group who did not undergo surgery by 24 months, 15 (10%) were on a surgical waiting list, 131 (86%) were not on a surgical waiting list, and 7 (5%) withdrew from follow-up. Of the 64 participants in the cholecystectomy group, by 24 months 13 (20%) were on a surgical waiting list, 13 (20%) were not on a surgical waiting list, 5 (8%) withdrew from follow-up, and 33 (52%) declined surgery. Supplementary appendix 2, table S3 shows the baseline characteristics of those randomised to laparoscopic cholecystectomy who did and did not have surgery.

Table 2 shows the results for SF-36 bodily pain profile up to 18 months for the two treatment groups. At three months, the conservative management group had a higher SF-36 bodily pain score than the cholecystectomy group (lower scores indicating more bodily pain), whereas after three months the cholecystectomy group had a higher score. The area under the curve up for SF-36 bodily pain to 18 months was 46.8 for both groups, with no difference (mean difference 0.0, 95% confidence interval −1.7 to 1.7; P=1.00, table 2). Sensitivity and complete case analysis of the primary outcome showed similar results (see supplementary appendix 2, table S4). No treatment effect modification was found in subgroup analyses for sex, age, and ethnicity (see supplementary appendix 2, figure S1). The compliance analysis of area under the curve up to 18 months also showed no evidence of a difference between the two groups (see supplementary appendix 2, table S5).

**Table 2:** Primary outcome and quality of life secondary outcomes up to 18 months

	Mean (SD); No		Mean difference (95% CI); P value
	Conservative management (n=217)	Laparoscopic cholecystectomy (n=217)	
**Primary outcome: SF-36 bodily pain**			
Baseline	44.5 (11.7); 202	43.4 (11.2); 205	
3 months	44.6 (11.5); 176	42.6 (11.0); 174	
9 months	46.6 (11.4); 144	47.9 (12.7); 160	
12 months	48.6 (11.6); 156	49.0 (11.4); 149	
18 months	49.4 (11.7); 167	50.4 (11.6); 161	
SF-36 bodily pain AUC over 18 months	46.8 (8.8); 203	46.8 (8.7); 205	0.0 (−1.7 to 1.7); 1.00
**Secondary outcomes**			
SF-36 bodily pain AUC over 24 months	47.2 (8.6); 203	46.8 (8.7); 205	−0.1 (−1.8 to 1.6); 0.94
SF-36:			
Physical functioning:			
18 months	47.8 (10.4); 120	49.6 (10.0); 114	−2.1 (−4.0 to −0.1); 0.04
24 months	47.7 (10.4); 103	49.1 (10.9); 99	−1.8 (−3.9 to 0.2); 0.08
Role physical:			
18 months	47.7 (10.7); 121	48.5 (11.0); 114	−1.1 (−3.3 to 1.2); 0.36
24 months	46.5 (11.0); 103	47.8 (11.9); 99	−2.0 (−4.5 to 0.4); 0.10
General health:			
18 months	44.3 (10.9); 121	46.6 (11.0); 111	−2.0 (−3.9 to −0.1); 0.04
24 months	44.9 (10.5); 103	44.8 (11.3); 99	−0.9 (−2.9 to 1.1); 0.40
Vitality:			
18 months	45.6 (11.2); 121	48.8 (11.4); 113	−3.9 (−6.0 to −1.7); 0.00
24 months	46.3 (11.2); 102	47.0 (11.3); 99	−2.2 (−4.5 to 0.0); 0.06
Social functioning:			
18 months	46.2 (11.3); 119	47.8 (12.0); 111	−1.2 (−3.8 to 1.3); 0.34
24 months	45.9 (12.5); 101	45.0 (12.5); 97	0.6 (−2.1 to 3.3); 0.68
Role emotional:			
18 months	44.1 (12.7); 121	46.4 (12.5); 114	−3.2 (−5.8 to −0.6); 0.02
24 months	45.6 (12.0); 103	44.8 (12.9); 99	−0.5 (−3.3 to 2.2); 0.71
Mental health:			
18 months	45.9 (11.0); 121	48.1 (11.0); 112	−2.4 (−4.6 to −0.1); 0.04
24 months	46.6 (11.5); 103	45.3 (11.4); 99	−0.0 (−2.4 to 2.3); 0.98
PCS:			
18 months	47.8 (10.3); 117	49.3 (10.2); 108	−1.2 (−3.2 to 0.8); 0.24
24 months	47.2 (10.9); 100	48.7 (11.4); 97	−1.9 (−4.0 to 0.1); 0.07
MCS:			
18 months	44.9 (12.6); 117	47.8 (11.7); 108	−2.9 (−5.4 to −0.5); 0.02
24 months	45.9 (12.1); 100	44.4 (12.6); 97	0.2 (−2.3 to 2.8); 0.85
CSQ total:			
18 months	21.3 (21.0); 113	15.8 (19.7); 101	6.6 (1.9 to 11.3); 0.01
24 months	20.7 (20.1); 98	14.0 (17.0); 91	9.0 (4.1 to 14.0); <0.001
Persistent symptoms score*:			
18 months	23.1 (24.1); 117	17.4 (22.2); 106	6.7 (1.0 to 12.3); 0.02
24 months	23.1 (23.0); 101	15.1 (18.4); 95	10.1 (4.2 to 16.0); <0.001

AUC=area under the curve; CI=confidence interval; CSQ=condition specific questionnaire; MSC=mental component summary; PCS=physical component summary; SD=standard deviation.

* Derived from two CSQ domains, pain and dyspepsia. For SF-36 norm-based scores, a higher score indicates better quality of life. For Otago gallstones CSQ, a higher score (range 0-100) indicates higher symptom burden and therefore poorer quality of life.

The secondary outcome, area under the curve for SF-36 bodily pain up to 24 months, did not differ between the two groups (mean difference −0.1, −1.8 to 1.6; P=0.94, table 2). Some small differences were found at 18 months for the SF-36 norm based scores (apart from bodily pain), but these disappeared at 24 months, with none of the effect sizes clinically important (table 2). Results of the condition specific questionnaire at 18 months were worse in the conservative management group compared with cholecystectomy group (mean difference 6.6, 95% confidence interval 1.9 to 11.3; P=0.01). At 24 months, results for the condition specific questionnaire were still higher in the conservative management group compared with cholecystectomy group. Similar results were observed for persistent symptoms. Supplementary appendix table S6 shows details of the outcomes at 3, 9, and 12 months.

NHS adjusted mean costs (using the resource data combined with the national cost data for the financial year 2019/20) were higher for cholecystectomy than for conservative management (£2510 *v* £1477 per participant), resulting in an adjusted cost difference of −£1033 (−$1334; −€1205) (95% credible interval −£1413 to −£632). Mean adjusted QALYs per participant were 1.413 for the cholecystectomy group and 1.395 for the conservative management group, with an adjusted mean difference of −0.019 (95% credible interval −0.06 to 0.02) for the 24 month follow-up period. Therefore, the incremental cost effectiveness ratio between conservative management and cholecystectomy was £55 235. At a cost effectiveness threshold of £20 000 per QALY there is a 0.94 probability of conservative management being cost effective. Moving from the standard practice of laparoscopic cholecystectomy to conservative management would on average result in lower costs and QALYs, with a saving of £55 235 per QALY forgone.

In the conservative management group, 32 (15%) participants had a complication by 18 months compared with 44 (20%) participants in the cholecystectomy group (relative risk 0.72, 95% confidence interval 0.46 to 1.14; P=0.17, table 3). The conservative management group experienced 25 (12%) pre-surgery complications and the cholecystectomy group 11 (5%), with most being cholecystitis or biliary colic. During surgery, nine (4%) complications occurred in the conservative management group and 24 (11%) in the cholecystectomy group, with most being bile or stone spillage from the gallbladder. Two (1%) complications occurred in the conservative management group and three (1%) in the cholecystectomy group within 30 days of discharge. After 30 days of discharge, complications occurred in one (0.5%) participant from each group. By 18 months, one (0.5%) cardiovascular death occurred in the cholecystectomy group. At 24 months, two additional complications occurred in the cholecystectomy group (see supplementary appendix table S7).

**Table 3:** Secondary outcome–complications up to 18 months. Values are number unless stated otherwise

	Conservative management (n=217)	Laparoscopic cholecystectomy (n=217)
No (%) of participants	32 (15)*	44 (20)*
No of complications:		
1	18	31
2	8	5
3	4	8
4	2	-
**Presurgery complications**		
No (%) of participants	25 (12)	11 (5)
No of complications:		
1	20	9
2	4	-
3	1	1
4	-	1
Types of complications:		
Cholecystitis	14	8
Biliary colic	8	2
Pancreatitis	2	3
Choledocholithiasis	2	-
Cholecystitis and jaundice	1	-
Choledocholithiasis and pancreatitis	1	-
Cholecystitis, choledocholithiasis, and jaundice	-	1
Cholecystitis and pancreatitis	1	-
Bouveret syndrome†	1	-
Cholecystitis, choledocholithiasis and pancreatitis	-	1
Jaundice	-	1
Right upper quadrant pain	1	-
**Intraoperative complications**		
No (%) of participants	9 (4)	24 (11)
No of complications:		
1	8	23
2	1	1
Types of complications:		
Bile/stone spillage from gallbladder	6	16
Injury to abdominal viscera (including liver tear or laceration)	1	5
Bleeding >500 mL	1	2
Bile leak from the bile duct, hepatic duct, or ducts at base of liver	1	1
Injury to bile duct	1	-
Ruptured empyema	-	1
**Postoperative complications**		
No (%) of participants	7 (3)	14 (6)
No of complications:		
1	5	9
2	1	4
3	1	1
Types of complications:		
Bleeding >500 mL	1	2
Bile leak requiring no treatment	2	3
Bowel obstruction:		
No treatment required	1	3
Surgery	-	1
Wound infection	2	-
Intraperitoneal: collection/abscess:		
No treatment required	1	3
Percutaneous drainage	1	-
Vomiting	-	3
Dizziness and hypotension	1	-
Haematoma	-	1
Missed stone in bile duct	-	1
Renal failure	-	1
Inflammation of residual gallbladder	1	-
Wound dehiscence	-	1
Complications <30 days of discharge:		
No (%) of participants	2 (1)	3 (1)
Cholangitis	-	1
Surgical site infection	1	1
Bile leak	-	1
Post-cholecystectomy syndrome‡	1	-
Complications >30 days of discharge:		
No (%) of participants	1 (0.5)	1 (0.5)
Right upper quadrant pain	-	1
Incisional hernia	1	-
Death - cardiovascular event:		
No (%) of participants	-	1 (0.5)

CI=confidence interval.

Relative risk of complications in the conservative management versus cholecystectomy group at 18 months was 0.72 (95% CI 0.46 to 1.14); P=0.17.

* One participant in each arm had their surgery converted from laparoscopic to open surgery. This was not a pre-defined complication within the C-Gall study.

† Bouveret’s syndrome occurs when a gallstone enters the small bowel through a bilioenteric fistula and is impacted in the duodenum or stomach, causing gastric outlet obstruction.

‡ Persistence of same symptoms reported by the patient.

By 18 months, nine of 200 (5%) participants in the conservative management group and 12 of 201 (6%) in the cholecystectomy group reported further treatment (relative risk 0.75, 95% confidence interval 0.31 to 1.78; P=0.51, table 4). The main treatments were analgesics, antibiotics, and endoscopic retrograde cholangiopancreatography. See supplementary appendix table S8 for details about further treatment at 24 months.

**Table 4:** Secondary outcome–further treatment up to 18 months. Values are number unless stated otherwise

	Conservative management (n=200)*	Laparoscopic cholecystectomy (n=201)*
No (%) of participants requiring at least one further treatment	9 (5)	12 (6)
No of treatments:		
1	7	8
2	2	2
3	-	1
7	-	1
Further treatment†:		
Pain relief	3	8
Antibiotics	2	3
ERCP	3	4
Antiemetic	1	-
Gas and air	1	-
Catheter for urinary retention	-	2
Bowel problem (unspecified)	-	1
Blood transfusion	-	1
Laparotomy washout and haemostasis	-	1
Fluids	-	1
Pancreatitis treatment	-	1
Unknown	1	-

CI=confidence interval; ERCP=endoscopic retrograde cholangiopancreatography.

Relative risk of further complications in the conservative management versus cholecystectomy group at 18 months was 0.75 (95% CI 0.31 to 1.78); P=0.51.

* Number followed-up.

† Corresponds to number of events.

## Discussion

The C-GALL trial is a multicentre, pragmatic trial to evaluate the clinical and cost effectiveness of conservative management compared with laparoscopic cholecystectomy to prevent recurrent symptoms and complications in adults with uncomplicated symptomatic gallstone disease in a secondary care setting. The trial was conducted across 20 secondary care sites in the UK NHS. The trial found no differences in overall bodily pain (primary outcome), quality of life, complications, or the need for further treatment between the two management strategies up to 18 months of follow-up. There was statistically significant evidence that gallbladder specific quality of life measures (condition specific questionnaire total score and condition specific questionnaire persistent symptoms)improved in the randomised cholecystectomy group at 18 months.

Before the trial, the C-GALL group envisaged that clinicians would be reluctant to recruit patients because of a bias towards surgery. Moreover, it was assumed that patients might be unwilling to consent to recruitment, as surgery is usually the only option discussed to relieve symptoms, and alternatives are probably not considered at both primary and secondary care level. During the trial, many patients opted for non-surgical treatment after the C-GALL team provided detailed information on the alternative option. Almost one third of patients randomised to receive conservative management subsequently received surgery, and 30% of those randomised to receive cholecystectomy had not undergone surgery by 24 months. It was also interesting to note the number of patients that opted not to take part in the trial, accepting conservative management over surgery during the trial when alternative treatments were discussed.

Prespecified sensitivity analyses showed that adherence to treatment allocation, missing data, and perceived potential impact of covid-19 (when national elective surgery was suspended for many months), did not change findings. Cost analysis showed that conservative management was less costly than cholecystectomy. The trial did not find a statistically significant difference in QALYs between the groups. The incremental cost effectiveness ratio was high, meaning important potential savings to the NHS with limited QALY loss by following a conservative management approach in the short term (up to 24 months). Longer term modelling suggested that a conservative management approach might be cost effective, but uncertainty was higher owing to limited information on subsequent surgeries in the randomised groups and quality of life beyond 24 months.

The within trial economic analysis indicated that intention to treat with conservative management was less costly than cholecystectomy over 24 months (mean difference £1033). Consistent with the primary outcome results of the C-GALL trial, a non-significant QALY difference of 0.019 favouring cholecystectomy was observed. The significant cost difference favouring conservative management and a small non-significant QALY difference favouring cholecystectomy resulted in an incremental cost effectiveness ratio of £55 235. That is, moving from the standard practice of intention to treat with laparoscopic cholecystectomy to conservative management would on average result in lower costs and QALYs, with a saving of £55 235 per QALY lost. Moreover, at a conventional cost effectiveness threshold of £20 000 per QALY used in the UK, the probabilistic analysis showed conservative management to have a 97% probability of being cost effective.^37^ Crucial to these results was the number of cholecystectomy procedures undertaken within the laparoscopic cholecystectomy group, which explained most of the cost difference between the trial groups. Participants crossing over from conservative management to cholecystectomy may erode the cost effectiveness of conservative management over a time horizon longer than the 24 month follow-up in the current trial. Therefore, extrapolation is clearly required beyond the trial follow-up with longer term follow-up of participants in the C-GALL trial.

### Strengths of this study

The strengths of the C-GALL trial include the pragmatic randomised controlled design and methodological rigour. The benefit of the sample size is reflected in the precision with which outcomes were estimated. The multicentre nature of the trial improves confidence in the generalisability of findings to the NHS. The recruited sample had a mean age of 50-51 years (slightly older than those who declined to take part (mean 48 years)). Among the population sample, participants were predominantly female (79%), white (86%) with Asian/Asian British and black/African/Caribbean/ black British participants comprising 7% and 3% of the sample, respectively. This is similar to national statistics for England and Wales for ethnicity (86%, 9%, and 3%, respectively),^39^ hence the study sample was representative of the general UK population. We believe that the population sample will be representative of adults presenting with uncomplicated symptomatic gallstone disease in the UK; however, we collected limited clinical data at baseline to confirm this.

This trial was pragmatic, where patients in the UK may not always receive the treatment they are offered and waiting lists for surgical treatment exist. We carefully tracked treatment after randomisation and monitored adherence. A major strength of the trial was the inclusion of sensitivity analyses, with adherence analysis, imputation for missing data, and potential impact of the covid-19 pandemic. Our findings remained unchanged after these analyses.

The study was designed as a superiority trial rather than a non-inferiority trial. We considered the study to be, in essence, a de-adoption study (removing cholecystectomy). When considering de-adopting cholecystectomy, the NHS would need strong, clear evidence that surgery is superior or inferior to conservative management. Performing the study to show that conservative management was non-inferior to surgery would be unlikely to provide strong enough evidence to change surgical practice. The randomised controlled design allowed unbiased, prospective collection of data on resource use and quality of life for comparable groups. This is a strength of the cost effectiveness analysis.

### Limitations of this study

An unexpected problem was the longer than expected time for patients on the surgical waiting list who had been allocated laparoscopic cholecystectomy. When designing the trial, it was anticipated that the waiting time would be, on average, six months. Therefore 18 months was chosen as the primary outcome follow-up time to reflect a time equivalent to 12 months after surgery. During the trial, however, we observed that patients often experienced longer waiting times for surgery, initially due to limited NHS resources, later compounded by the impact of covid-19 related restrictions. To address this, we added a 24 month follow-up point. Our sensitivity analyses on adherence to treatment suggested that the waiting list was unlikely to bias the study findings. The waiting list may, however, limit generalisability to some other countries’ jurisdictions. A further limitation was the non-blinding of participants and treating surgeons to allocation. In this trial, the pragmatic research question tested the most effective treatment strategy in a real life setting, leading to an inevitable lack of blinding. Finally, the cost effectiveness analysis was conducted from an NHS perspective, which may impact the generalisability of its results to other healthcare settings.

### Comparison with other studies

The results of the C-GALL trial add to existing evidence. Two small Norwegian randomised controlled trials with a total of 201 participants found that 55% of people randomised to observation did not require an operation during the 14 year follow-up period, and 12% of people randomised to cholecystectomy did not undergo the scheduled procedure.^40^ This contrasts with 70% randomised to conservative management not undergoing surgery at 24 months in the current trial and 30% in the surgery group not undergoing cholecystectomy.

The SECURE (restrictive strategy versus usual care for cholecystectomy in patients with gallstones and abdominal pain) trial^41^ was a non-inferiority multicentre randomised controlled trial in the Netherlands to assess the effects of immediate cholecystectomy versus a restrictive strategy. Participants underwent cholecystectomy only when they fulfilled five prespecified surgery criteria at clinic visits. The authors reported that 7.7% fewer patients had cholecystectomies with the restrictive strategy, but 37% in both groups continued with abdominal pain. The investigators concluded that the current surgical management of patients with gallstone disease and abdominal symptoms is suboptimal, that a restrictive policy is not a solution, and that doctors need to be more careful in advising a surgical approach to patients with symptoms of gallstone disease. The findings of the C-GALL trial are consistent with the conclusions of the SECURE trial and provide stronger evidence from a broader range of patients, as the current trial enrolled participants with biliary colic or acute cholecystitis (SECURE only focused on biliary colic).

### Policy implications

Current clinical guidelines recommend laparoscopic cholecystectomy for biliary pain or acute cholecystitis and radiological evidence of gallstones.^42^ Hence, laparoscopic cholecystectomy remains the first line treatment for people with symptomatic gallstone disease and is one of the most common elective surgical procedures performed in the NHS.^43^ The C-GALL trial found that in adults presenting with uncomplicated symptomatic gallstone disease to secondary care, conservative management may be effective and cost effective than surgery. The study found it is safe to manage patients conservatively for at least 18 months. The crossover between groups suggested that it remains key to identifying patients who require surgery. As healthcare professionals often underestimate surgical risks,^44^ and post-cholecystectomy syndrome may occur, a discussion about conservative management should form part of a patient’s decision making and consent process.

### Conclusion

In the short term (<18 months), conservative management, as an alternative to surgery, may be effective and cost effective for patients with uncomplicated symptomatic gallstone disease.

We conclude that the costs and benefits will continue to be incurred in both groups beyond 24 months, so future research should focus on long term follow-up data to establish lifetime cost effectiveness and aid identification of the cohort of patients who will benefit from surgery.

**What is already known on this topic**

Two previous, small randomised trials showed that cholecystectomy remains the treatment of choice for many people with symptomatic gallstone disease
However, about half of the people in the observation groups might not require surgery or develop complications in the long term
Evidence suggested that a conservative treatment approach may represent an alternative to surgery

**What this study adds**

In the short term (<18 months), conservative management, as an alternative to surgery, may be effective and cost effective in patients with uncomplicated symptomatic gallstone disease
Costs and benefits will continue to be incurred in both trial groups beyond 24 months
Future research is needed to collect long term follow-up data to establish lifetime cost effectiveness and to identify those patients who will benefit from surgery

## Data Availability

The datasets generated during the study will be available upon reasonable request from the corresponding author.

## Web extra

Extra material supplied by authors

Supplementary information: Appendices 1 and 2

## Notes

Contributors: IA, RH, KG, AA, MB, BC, PM, SW, and CR conceived the trial. IA, KI, RH, KG, AA, BC, JB, PM, CR designed the trial. IA was involved in recruitment and was responsible for the day-to-day oversight of the trial. KI, RB, VB, and CR were responsible for the day-to-day management of the trial. JH conducted the statistical analysis. RH conducted the health economics analysis. SC contributed to oversight of the day-to-day management of the trial. BC was responsible for the conduct of the trial, recruitment, and follow-up of participants, and focused on the patient and public involvement components of the trial. MF contributed to the design of the trial software and data curation. GM contributed to the statistical analysis. IA, KI, JH, RH, KG, RB, VB, AA, JB, MB, SC, BC, MF, GM, PM, SW, and CR contributed to the interpretation of findings and the reporting of the study. All authors have critically reviewed and approved the final manuscript version. The corresponding author had full access to all the data in the trial and had final responsibility for the decision to submit for publication. IA and CR act as guarantors. The corresponding author attests that all listed authors meet authorship criteria and that no others meeting the criteria have been omitted.

The study guarantors (IA and CR) affirm that the manuscript is an honest, accurate, and transparent account of the study being reported, that no important aspects of the study have been omitted; and that any discrepancies from the study as planned (and, if relevant, registered) have been explained.

Dissemination to participants and related patient and public communities: In collaboration with our patient and public involvement partners and patient and public involvement group, we plan to develop dissemination materials (including an infographic) for participants. We will prepare a plain-language summary for the C-GALL website. We will share our findings with relevant charity and support groups, including gutsuk! (https://gutscharity.org.uk/).

Provenance and peer review: Commissioned; not externally peer reviewed.
