# Supplementary material for: Effectiveness of conservative management versus laparoscopic cholecystectomy in the prevention of recurrent symptoms and complications in adults with uncomplicated symptomatic gallstone disease (C-GALL trial): pragmatic, multicentre randomised controlled trial
Source: BMJ. 2023 Dec 6;383:e075383. doi: 10.1136/bmj-2023-075383 (PMC10698555; doi:10.1136/bmj-2023-075383)
Supplement: Supplementary file 1 — Supplementary information: Appendices 1 and 2 [file ahmi075383.ww.pdf]

Appendix 1

A randomised controlled trial comparing laparoscopic **c**holecystectomy with observation/conservative management for preventing recurrent symptoms and complications in adults with uncomplicated symptomatic **gall**stones. (C-GALL trial)

Statistical Analysis Plan

Version:	1.0
Version Date:	
Protocol Version:	11.0
Protocol Date:	6th July 2020

Co-Sponsors

Name:	University of Aberdeen
Address:	Research Governance Office, Room 7, Foresterhill Annexe, Foresterhill, Aberdeen, AB25 2ZD
Name:	Grampian Health Board
Address:	Research and Development Office, Foresterhill House Annexe, Foresterhill, Aberdeen AB25 2ZB
Sponsor number	01.014.16

Investigators

Clinical Chief Investigator

Name:	Prof Irfan Ahmed

Co-Chief Investigator

Name:	Prof Craig Ramsay

Funder

Name:	NIHR Health Technology Assessment (HTA) Programme
Funder number:	14/192/71
Funder start date:	1 April 2016
Funder end date:	31 March 2022

Other

ISRCTN:	55215960
REC number:	16/NS/0053

Signatures

Clinical Chief Investigator
Prof Irfan Ahmed
Date:

Co- Chief Investigator
Prof Craig Ramsay
Date:

Senior Statistician
Dr Lorna Aucott
Date:

Trial Statistician
Miss Jemma Hudson
Date:

Amendment History

SAP version	Protocol version	Section number changed	Description of and reason for change	Date changed

Glossary of Abbreviations

AE	Adverse Event
AUC	Area Under the Curve
CHaRT	Centre for Healthcare Randomised Trials
CI	Confidence Interval
CRF	Case Report Form
CSQ	Otago Gallstones Condition-Specific Questionnaire
DMC	Data Monitoring Committee
HSRU	Health Services Research Unit
ITT	Intention-to-Treat
SAE	Serious Adverse Event
SAP	Statistical Analysis Plan
SD	Standard Deviation

Table of Contents

1 Introduction 8

2 Study Aims and Objectives 8

3 General Study Design 8

4 Interventions to be evaluated 8

5 Randomisation, Allocation and Blinding 9

6 Outcome Measures 9

6.1 Primary Outcome 9

6.2 Secondary Outcomes 9

7 Timing of Outcome Measurements 10

8 Adverse Events 10

9 Sample Size and Power Calculation 10

10 Statistical Methods 11

10.1 General Methods 11

10.2 Interim Analysis 11

10.3 Primary Outcome 11

10.4 Secondary Outcomes 11

10.5 Subgroup Analysis 12

10.1 Compliance 12

10.2 Missing Data 12

10.2.1 Missing Outcome Data 12

10.2.2 Missing Baseline Data 12

10.3 Statistical software 13

11 COVID-19 13
12 Dummy Tables 13
13 Dummy Figures 22
14 References 25
15 Appendix 25
15.1 Derived Patient Reported Outcome Measures (PROMs) 25
15.1.1 References 26
15.2 Stata code 26

Table of Tables

Table 1 Overall Recruitment and by centre 13
Table 2 Reasons why participants were not randomised 14
Table 3 Baseline Characteristics 14
Table 4 Surgical Procedure up to 24 months 15
Table 5 Primary Outcome - AUC SF-36 Bodily Pain up to 18 months 15
Table 6 AUC SF-36 Bodily Pain up to 24 months 16
Table 7 Secondary Outcomes - quality of life 17
Table 8 Secondary Outcomes – complications for 18 and 24 months 19
Table 9 Secondary Outcomes - further treatment up to 18 and 24 months 19
Table 10 Other Appointments up to 18 and 24 months 20
Table 11 Medication prescribed up to 18 and 24 months 20
Table 12 Serious Adverse Events up to 18 and 24 months 20
Table 13 Crossovers up to 24 months 20
Table 14 End of trial status up to 24 months 21
Table 15 Withdrawals and post-randomisation exclusions up to 24 months 21
Table 0.1 Calculation of PROMs scores 26
Table 0.2 Stata code 27

Table of Figures

Figure 1 Recruitment graph 23
Figure 2 Participant flow 23
Figure 3 SF-36 Bodily pain over time 24
Figure 4 Subgroup analysis 24

Introduction

This statistical analysis plan (SAP) documents the analysis for the C-GALL trial, a randomised controlled trial comparing laparoscopic cholecystectomy with observation/conservative management for preventing recurrent symptoms and complications in adults with uncomplicated symptomatic gallstones. The SAP is based on the protocol version 11 and any deviations from the plan will be described.

Study Aims and Objectives

The **primary aim** of the study is to assess the clinical and cost-effectiveness of observation/conservative management compared with cholecystectomy for preventing recurrent symptoms and complications in adults presenting with uncomplicated symptomatic gallstones in a secondary care setting.

The **primary patient objective** is to compare medical management with cholecystectomy in terms of participants' quality of life using the SF-36 short-form health survey bodily pain domain at up to 18 months after randomisation.

The **secondary objectives** are to compare medical management with surgical treatment (cholecystectomy) in terms of condition specific quality of life; SF-36 domains (excluding bodily pain domain); complications; need for further treatment; persistent symptoms; health care resource use; costs. Secondary outcomes are at 18 and 24 months after randomisation. SF-36 short-form health survey bodily pain domain at up to 24 months after randomisation will also be reported.

General Study Design

A pragmatic, multi-centre parallel group patient randomised superiority trial (with internal pilot phase) to test if the strategy of standard cholecystectomy is more (cost-) effective than observation/conservative management at 18 months post-randomisation.

The null hypothesis being tested is that there is no difference between medical management and cholecystectomy. The alternative hypothesis is that cholecystectomy is superior.

Interventions to be evaluated

Laparoscopic cholecystectomy: this is the current standard surgical procedure for the management of symptomatic gallstone disease. The gall bladder is removed with the stones within it using keyhole techniques (laparoscopy).

Medical management: this involves the prescription of analgesics to relieve the biliary pain along with generic lifestyle advice. In the long-term, it may also include advice to eat a healthy diet with regular meals.

Randomisation, Allocation and Blinding

All participants who agree to enter the study will be logged with the central trial office and given a unique Study Number. Randomisation will utilise the existing proven remote automated computer randomisation application in the central trial office in the Centre for Healthcare Randomised Trials (CHaRT, a fully registered UK CRN clinical trials unit) in the Health Services Research Unit (HSRU), University of Aberdeen.

Participants are randomised 1:1 to receive either laparoscopic cholecystectomy or observation/conservative management. A random element will be incorporated into the randomisation algorithm. Treatment allocation is minimised by centre, gender and age (<35; 35-64; ≥ 65).

The trial statistician will not be blinded to treatment allocation.

Outcome Measures

Primary Outcome

The primary patient outcome measure will be quality of life as measured by area under the curve (AUC) up to 18 months post-randomisation using the SF-36 bodily pain domain (AUC measures at 3, 9, 12 and 18 months). The SF-36[1] is a generic measure obtained from a multi-purpose, short-form health survey with 36 questions. It has 36 items that is combined into 8 health domain scales (including Bodily Pain) and then further into two Summary measures.

Secondary Outcomes

The secondary outcome measures will include:

Otago Gallstones Condition-Specific Questionnaire (CSQ)

SF-36 health domains scales (excluding bodily pain domain)

Complications

Need for further treatment

Persistent symptoms

AUC up to 24 months post-randomisation for SF-36 bodily pain

The CSQ[2] is a condition specific quality of life measure with four domains: Physical Functioning (pain, dyspepsia and diet changes), Systemic Functioning (fatigue), Social Functioning (daily duties, leisure,

relationships) and Emotional Functioning (mood). It contains 12 items, each with a 5-point Likert response scale.

Complications will be defined as any intra- operative and post-operative complications.

Need for further treatment will be patient reported.

Persistent symptoms will be patient reported and will consist of two sections (pain and dyspepsia) of the CSQ.

Timing of Outcome Measurements

	**Baseline**	**Surgery[1]**	**3 months**	**9 months**	**12 months**	**18 months**	**6 monthly thereafter**
SF-36	X		X	X	X	X	X
CSQ	X		X	X	X	X	X
Complications	X	X	X	X	X	X	X
Need for further treatment			X	X	X	X	X

[1]Surgery (and post-surgery) CRF completed where appropriate.

CRF Case Report Form.

All times points are after randomisation.

Adverse Events

Each initial Adverse event (AE) will be considered for severity, causality or expectedness. A serious adverse event (SAE) is any AE that:

Results in death

Is life-threatening

Requires hospitalisation, or prolongation of existing inpatients' hospitalisation

Results in persistent or significant disability or incapacity Is a congenital anomaly or birth defect is otherwise considered medically significant by the investigator.

Please see the Study Protocol for more details on AEs. The number of Adverse events (AEs) and serious adverse events (SAEs) and the proportion of participants with an event will be presented. These will be tabulated and not analysed and will be summarised by Intention-to-Treat (ITT) as well as, as treated.

Sample Size and Power Calculation

The primary outcome is AUC of the SF-36 bodily pain domain up to 18 months post-randomisation. A study with 194 participants per group (388 in total) would have a 90% power at a 5% significance level to detect a difference of 0.33 standard deviation (SD). A total sample size of 430 participants to allow for 10% of complete missing outcome data.

Statistical Methods

General Methods

All the main analysis will be based on the ITT principle. The results of the trial will be presented following the standard CONSORT recommendations[3]. Baseline and follow-up data will be summarised using appropriate descriptive statistics and graphical summaries. Treatment effects will be presented with 95% confidence intervals (CIs) (apart from subgroup analysis where a stricter level will be used). There will be no adjustment to secondary outcomes CIs for multiple testing. All eligible participants who provided consent will be included in the analysis. Any post-randomisation exclusions will be removed. See Appendix (section 0) for how patient reported outcome measures (PROMs) are derived along with the sample Stata code for the analysis of the outcomes.

Interim Analysis

There are no planned interim analyses for efficacy or futility but an independent Data Monitoring Committee (DMC) will monitor trial progress and specifically any safety issues. Analysis will take place after full recruitment and follow-up.

Primary Outcome

The primary outcome, AUC SF-36 bodily pain domain up to 18 months will be analysed using linear regression with adjustment for the minimisation covariates (gender, age and including site as a random effect). The AUC for each participant will be generated by the trapezium rule. An SF-36 bodily pain profile will be presented graphically and in tabular form. If there is missing baseline data, then the centre specific baseline mean will be used.

Our primary analysis will include all participants that have at least one time point up to 30 months post-randomisation. For those participants with data missing at the 18 months time point, multiple imputation will be used to impute their score. Secondary analysis of the primary outcome will be performed on those participants that have an 18 months score. A sensitivity multiple imputation analysis will be performed using data only up to 18 months.

Secondary Outcomes

For the secondary outcome, SF-36 bodily pain AUC up to 24 months post-randomisation will be analysed in a similar way to the primary outcome. All other secondary outcomes will be analysed with generalised models appropriate for the distribution of the outcome with adjustment for the minimisation covariates (gender, age and including site as a random effect) up to 18 and 24 months.

SF-36 domains (excluding bodily pain), CSQ and persistent symptoms will be analysed using repeated measures including baseline score as an additional covariate. Treatment effects will be estimated from time-by-treatment interactions at each time point. P-values will only be presented at 18 and 24 months post-randomisation. Data collected after 24 months will be summarised descriptively, but no formal statistical test will be used.

Subgroup Analysis

Planned subgroup analyses are intended to explore potential effect modifications of gender (male, female), age (<35; 35-64; ≥ 65) and ethnicity using the UK census ethnic groupings (white; mixed/multiple ethnic groups; Asian/Asian British; Black/African/Caribbean/Black British; Other) or a similar grouping depending on the data. Subgroup by treatment interactions will be assessed by including interaction terms in the models outlined above.

A stricter level of statistical significance (2-sided 1% significance level) will be applied to these analyses given their exploratory nature. Corresponding 99% confidence intervals will therefore be calculated.

Compliance

We will explore the influence of compliance on the treatment effect for the primary outcome by doing a per-protocol analysis and if appropriate a complier adjusted causal estimation (CACE) will be explored. Compliance will be defined as participants who receive their allocated treatment within 24 months. For the laparoscopic cholecystectomy group, participants who receive emergency laparoscopic cholecystectomy will be defined as non-compliant.

Missing Data

Missing Outcome Data

The sensitivities of all treatment effect estimates to missing outcome data will be explored for the primary outcome only; these models will explore the robustness of the treatment estimates. We will follow the strategy outlines in White[4]. The analysis will use all available data that we believe are valid under the assumption of missing at random. We will then use a suite of sensitivity analysis to explore the robustness of the primary analysis to departures from assumptions, including all randomised participants. If required, sensitivity analyses will include but is not limited to multiple imputation, imputing a range of values for missing data under missing not at random assumptions as well as imputing data assuming a linear trend.

Missing Baseline Data

Data missing at baseline will reported as such. If required for models for primary and/or secondary outcomes continuous data will be imputed with the centre specific mean of that variable, missing binary/categorical data will include a missing indicator [5].

Statistical software

All analysis will be carried out in Stata 16[6] (or the current version available).

COVID-19

The effect of COVD-19 will be explored. In the first instance, periods before and after COVID-19 will be summarised using appropriate descriptive statistics and graphical summaries. If need be, formal analysis will be carried out to explore the effect of COVID-19.

Dummy Tables

Table 1 Overall Recruitment and by centre

Centre	Patients randomised N=	Randomised to Medical management N=	Randomised to Surgery management N=
Overall recruitment			
Recruitment by centre			
Aberdeen			
Nottingham			
Taunton (Somerset) Royal Free (London)			

Values are n (%)

Table 2 Reasons why participants were not randomised

	N=
Reasons for ineligibility	
Unable to consent	
Pregnancy	
A history of acute pancreatitis	
...	
Reasons for declining to take part	
Did not want to be randomised	
Treatment preference for medical management	
Treatment preference for surgical management...	

Values are n

Table 3 Baseline Characteristics

	**Medical Management N=**	**Surgical Management N=**
Age (years)		
Gender		
Male		
Female		
Ethnicity		
White		
Mixed/ Multiple ethnic groups		
Asian/ Asian British		
Black/ African/ Caribbean/ Black British		
Chinese		
Arab		
Other		
BMI (kg/m^2)		
Diabetes Diagnosis		
No		
Type I		
Type II		
Gallbladder wall		
Normal		
Thick		
Not recorded		
Thickness of gallbladder wall		
Hypertension		
Yes		
No		
SF-36		
Bodily pain		
Physical Functioning		
Role Physical		
General Health		
Vitality		

Social Functioning		
Role Emotional		
Mental Health		
CSQ Total		
Persistent symptoms		

Values are n (%) or mean (standard deviation); number who provided data.

Table 4 Surgical Procedure up to 24 months

	Medical Management N=	Surgical Management N=
Received Surgery		
Yes		
No		
	**Received surgery n=**	**Received surgery n=**
Time to surgery (weeks)		
Elective surgery		
Procedure Type		
Laparoscopic		
Open		
Laparoscopic converted to open		
Reasons for conversion		
...		
Grade of operating surgeon		
Consultant		
Registrar		
Specialty (SAS grade)		
SHO		
Specialist trainee		
Other		
Prophylactic antibiotic used in the operation		
Operation time (minutes)		
Difficulty of surgery		
Straightforward		
Mildly difficult		
Moderately difficult		
Length of hospital stay (days)		
**Histopathology**		
Normal gallbladder		
Cholecystitis		
Acute		
Chronic		
No		
Incidental biliary cancer		

Values are n (%) or mean (standard deviation); number who provided data.

Table 5 Primary Outcome - AUC SF-36 Bodily Pain up to 18 months

	Medical Management	Surgical Management	MD[1]	95% CI	p-value

	N=	N=			
Baseline					
3 months					
9 months					
12 months					
18 months					
AUC up to 18 months					

AUC Area Under the Curve; CI Confidence Interval; [1]MD Mean Difference.

MD, CI and p-value will only be presented for AUC.

Values are mean (standard deviation) number who provided data.

Table 6 AUC SF-36 Bodily Pain up to 24 months

	Medical Management N=	Surgical Management N=	MD[1]	95% CI	p-value
Baseline					
3 months					
9 months					
12 months					
18 months					
24 months					
AUC up to 24 months					

AUC Area Under the Curve; CI Confidence Interval; [1]MD Mean Difference.

MD, CI and p-value will only be presented for AUC.

Values are mean (standard deviation) number who provided data.

Table 7 Secondary Outcomes - quality of life

	Medical Management N=	Surgical Management N=	MD[1]	95% CI	p-value[2]
SF-36					
Physical Functioning					
Baseline					
3 months					
9 months					
12 months					
18 months					
24 months					
Role Physical					
Baseline					
3 months					
9 months					
12 months					
18 months					
24 months					
General Health					
Baseline					
3 months					
9 months					
12 months					
18 months					
24 months					
Vitality					
Baseline					
3 months					
9 months					
12 months					
18 months					
24 months					
Social Functioning					
Baseline					
3 months					
9 months					
12 months					
18 months					
24 months					
Role Emotional					
Baseline					
3 months					
9 months					
12 months					
18 months					
24 months					
Mental Health					
Baseline					
3 months					
9 months					
12 months					
18 months					
24 months					

CSQ Total	
Baseline	
3 months	
9 months	
12 months	
18 months	
24 months	
Persistent symptoms	
Baseline	
3 months	
9 months	
12 months	
18 months	
24 months	

[1] MD Mean Difference.

[2] only be presented for 18 and 24 months.

CI Confidence Interval.

Values are n; mean (standard deviation).

Table 8 Secondary Outcomes – complications for 18 and 24 months

	Medical Management N=	Surgical Management N=
Number of participants with a complication		
RR[1] (95% CI); p-value		
Number of complications		
**Details**		
Intra-operative complications		
Bleed >500ml		
Injury to abdominal viscera		
Bile leak from the bile duct, hepatic duct or ducts at base of liver		
Bile/stone spillage from gall bladder		
Injury to bile duct		
Post-operative complications		
Bleed >500ml		
Injury to adnominal viscera		
Injury to bile duct		
Bowel obstruction		
No treatment		
Surgery		
Bile leak		
Per-cutaneous drainage		
No treatment		
Surgery		
Surgical Site Infection		
Wound infection		
Intraperitoneal - collection/abscess		
Per-cutaneous drainage		
No treatment		
Surgery		
Thrombi-embolic complications		
Deep vein thrombosis		
Pulmonary embolism		
Pain-requiring additional analgesia		
Urinary retention		
Other		

[1] RR Relative Risk.

CI Confidence Interval.

Values are n (%).

Table 9 Secondary Outcomes - further treatment up to 18 and 24 months

	Medical Management N=	Surgical Management N=
Number of participants		
RR[1] (95% CI); p-value		
Details of Treatment		
…		

[1] RR Relative Risk.

CI Confidence Interval.

Values are n (%).

Table 10 Other Appointments up to 18 and 24 months

	Medical Management N=	Surgical Management N=
Number of participants who required appointments		
**Details**		
Visited NHS hospital outpatients		
Visited NHS hospital A&E		
Visited GP		
Appointment with care provider		

Values are n (%).

Table 11 Medication prescribed up to 18 and 24 months

	Medical Management N=	Surgical Management N=
Number of participants prescribed medication		
Type		
Paracetamol		
Anti-inflammatory		
Codeine		
Opiate		
Buscopan		
Other		

Values are n (%).

Table 12 Serious Adverse Events up to 18 and 24 months

	Medical Management N=	Surgical Management N=
Number of participants who experienced an SAE		
Number of SAE's		
**Details**		
Life-threatening		
Hospitalisation		
Death		
…		

Values are n (%).

Table 13 Crossovers up to 24 months

	Medical Management N=	Surgical Management N=
Number of crossovers		
Requested by		
Participant		
Clinician		
Other		
Reason for crossover for those requested by participant		
…		
Reason for crossover for those requested by clinician		
…		
Reason for crossover for those requested by other		
…		
Time since randomisation when crossover occurred		

Values are n (%).

Table 14 End of trial status up to 24 months

	Medical Management N=	Surgical Management N=
Had surgery		
Elective		
Emergency		
On waiting list		
On medical management		

Values are n (%).

Table 15 Withdrawals and post-randomisation exclusions up to 24 months

	Medical Management N=	Surgical Management N=
Number of withdrawals		
**Reasons**		
…		
Number of post-randomisation exclusions		
**Reasons**		
…		

Values are n (%).

Dummy Figures

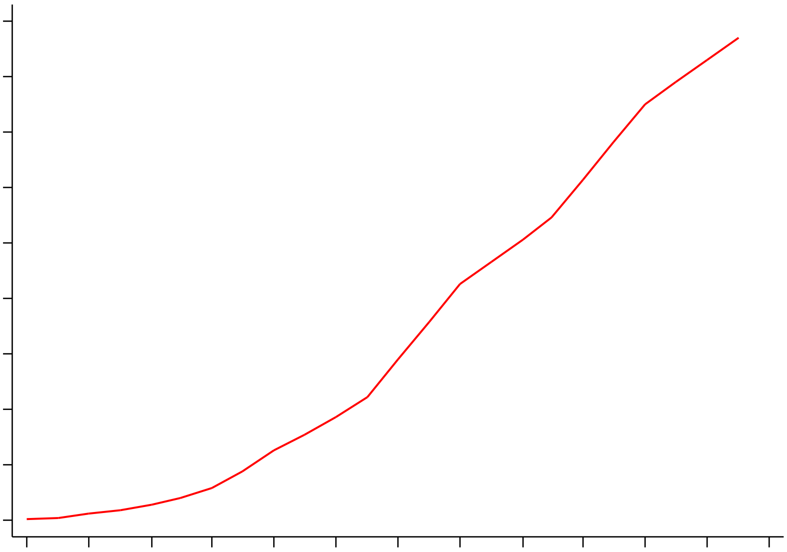

Figure 1 Recruitment graph

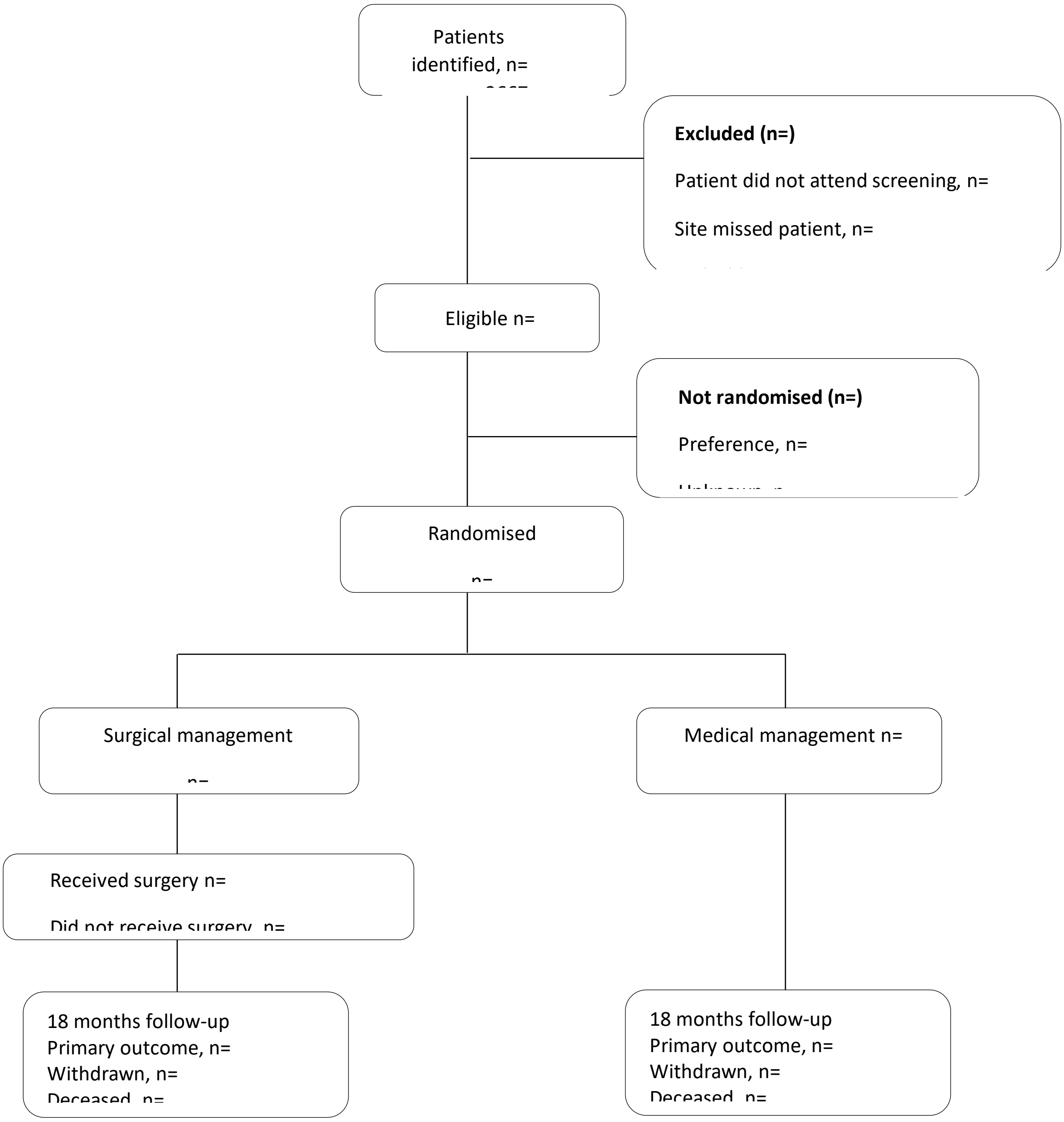

Figure 2 Participant flow

Figure 3 SF-36 Bodily pain over time

Figure 4 Subgroup analysis

References

1. Ware JE, Gandek B. Overview of the SF-36 health survey and the international quality of life assessment (IQOLA) project. *J Clin Epidemiol*. 1998;51(11):903-912. http://www.sciencedirect.com/science/article/pii/S089543569800081X. doi: https://doi.org/10.1016/S0895-4356(98)00081-X.

2. Chen TYT, Landmann MG, Potter JC, Van Rij AM. Questionnaire to aid priority and outcomes assessment in gallstone disease. *ANZ J Surg*. 2006;76(7):569-574. https://doi.org/10.1111/j.1445-2197.2006.03777.x. doi: 10.1111/j.1445-2197.2006.03777.x.

3. Schulz KF, Altman DG, Moher D. CONSORT 2010 statement: Updated guidelines for reporting parallel group randomised trials. *BMJ*. 2010;340:c332. http://www.bmj.com/content/340/bmj.c332.abstract. doi: 10.1136/bmj.c332.

4. White IR, Horton NJ, Carpenter J, statistics, reader in medical and social, Pocock SJ. Strategy for intention to treat analysis in randomised trials with missing outcome data. *BMJ*. 2011;342:d40. http://www.bmj.com/content/342/bmj.d40.abstract. doi: 10.1136/bmj.d40.

5. Sullivan TR, White IR, Salter AB, Ryan P, Lee KJ. Should multiple imputation be the method of choice for handling missing data in randomized trials? *Stat Methods Med Res*. 2018;27(9):2610-2626. https://doi.org/10.1177/0962280216683570. doi: 10.1177/0962280216683570.

6. StataCorp. 2019. stata statistical software: Release 16. college station, TX: StataCorp LLC. .

7. Maruish, M. E. (Ed.). User's manual for the SF-36v2 health survey (3rd ed.). *Lincoln, RI: QualityMetric Incorporated*.

Appendix

Derived Patient Reported Outcome Measures (PROMs)

The PROMs are SF-36, CSQ and persistent symptoms. Codes developed in-house will be checked and validated by an independent statistician using dummy data.

describes how each score will be calculated.

Table 0.1 Calculation of PROMs scores

PROMs	Calculation
SF-36	SF-36 version2 will be calculated following the recommendations of the SF-36v2 manual[7].
CSQ*	CSQ will be calculated as follows which has been confirmed by the authors of the published paper[1]:<br>A numerical value of 0-4 is assigned to each response category in ascending order of symptom severity: leftmost option (not at all) = 0; rightmost option (extremely) = 4<br>The total score is calculated by adding up the score for each question. Please note that the last question (question 5) should be excluded<br>Total percentage score = Total score / Maximum possible score * 100<br>The total score ranges from 0-100 with a higher score indicating higher symptom burden and therefore poor quality of life.<br>If any question is not answered (i.e. there is missing data) then the score cannot be calculated.
Persistent symptoms	This is an unvalidated score and will be calculated as described for the CSQ measure but only using the relevant questions (see section 0)

*The Otago gallstones condition-specific questionnaire

References

1. Maruish, M. E. (Ed.). User's manual for the SF-36v2 health survey (3rd ed.). *Lincoln, RI: QualityMetric Incorporated*.

2. Chen TYT, Landmann MG, Potter JC, Van Rij AM. Questionnaire to aid priority and outcomes assessment in gallstone disease. *ANZ J Surg*. 2006;76(7):569-574. https://doi.org/10.1111/j.1445-2197.2006.03777.x. doi: 10.1111/j.1445-2197.2006.03777.x.

Stata code

provides sample Stata code for the analysis of each outcome.

Table 0.2 Stata code

Outcome	Stata code
**Primary outcome:**	
AUC up to 18 months post-randomisation for SF-36 bodily pain	mixed auc_18_months i.Medical i.AgeBand i.Gender || CentreNo:<br>auc_18_months continuous (area under the curve from randomisation up to 18 months)<br>Medical binary (coded 0 for laparoscpic cholecystectomy and 1 for observation/conservative management)<br>AgeBand categorical (<35 years; 35-64 years; ≥65 years)<br>Gender binary (0 for male and 1 for female)<br>CentreNo categorical (corresponds to each recruiting centre)<br>Treatment effect<br>lincomest _b[1.Medical]
**Secondary outcomes:**	
Otago Gallstones Condition-Specific Questionnaire	mixed csq csq_b i.AgeBand i.Gender i.Medical##i.TimePoint || CentreNo: || StudyNo:<br>csq is continuous<br>csq_b is continuous baseline score<br>AgeBand categorical (<35 years; 35-64 years; ≥65 years)<br>Gender binary (0 for male and 1 for female)<br>Medical binary (coded 0 for laparoscopic cholecystectomy and 1 for observation/conservative management)<br>TimePoint is a categorical variable corresponds to the follow-up time points (coded 3 = 3 months, 9 = 9 months, 12 = 12 months, 18 = 18 months and 24 = 24 months)<br>CentreNo categorical (corresponds to each recruiting centre)<br>StudyNo is a unique participant identifier<br><br>Treatment effect for 3 months<br>lincomest _b[1.Medical]<br><br>Treatment effect for 9 months<br>lincomest _b[1.Medical] + _b[1.Medical#9.TimePoint]<br><br>Treatment effect for 12 months<br>lincomest _b[1.Medical] + _b[1.Medical#12.TimePoint]<br><br>Treatment effect for 18 months<br>lincomest _b[1.Medical] + _b[1.Medical#18.TimePoint]<br><br>Treatment effect for 24 months<br>lincomest _b[1.Medical] + _b[1.Medical#24.TimePoint]

SF-36 domains (excluding bodily pain domain)	mixed sf_36 sf_36_b i.AgeBand i.Gender Medical##i.TimePoint || CentreNo: || StudyNo:<br>sf is continuous<br>sf_36_b is continuous baseline score<br>AgeBand categorical (<35 years; 35-64 years; ≥65 years)<br>Gender binary (0 for male and 1 for female)<br>Medical binary (coded 0 for laparoscopic cholecystectomy and 1 for observation/conservative management)<br>TimePoint is a categorical variable corresponds to the follow-up time points (coded 3 = 3 months, 9 = 9 months, 12 = 12 months, 18 = 18 months and 24 = 24 months)<br>CentreNo categorical (corresponds to each recruiting centre)<br>StudyNo is a unique participant identifier<br><br>Treatment effect for 3 months<br>lincomest _b[1.Medical]<br><br>Treatment effect for 9 months<br>lincomest _b[1.Medical] + _b[1.Medical#9.TimePoint]<br><br>Treatment effect for 12 months<br>lincomest _b[1.Medical] + _b[1.Medical#12.TimePoint]<br><br>Treatment effect for 18 months<br>lincomest _b[1.Medical] + _b[1.Medical#18.TimePoint]<br><br>Treatment effect for 24 months<br>lincomest _b[1.Medical] + _b[1.Medical#24.TimePoint]
Complications	mepoisson Complications i.Medical i.AgeBand i.Gender || CentreNo:, irr vce(robust)<br>Complications binary (0 for no complications and 1 for had complication(s))<br>Medical binary (coded 0 for laparoscopic cholecystectomy and 1 for observation/conservative management)<br>AgeBand categorical (<35 years; 35-64 years; ≥65 years)<br>Gender binary (0 for male and 1 for female)<br>CentreNo categorical (corresponds to each recruiting centre)<br>irr is incidence rate ratio<br>Treatment effect<br>lincomest _b[1.Medical]
Need for further treatment	mepoisson FurtherTreatment i.Medical i.AgeBand i.Gender || CentreNo:, irr vce(robust)

	FurtherTreatment binary (coded as 0 for no further treatment and 1 for had further treatment)<br>Medical binary (coded 0 for laparoscopic cholecystectomy and 1 for observation/conservative management)<br>AgeBand categorical (<35 years; 35-64 years; ≥65 years)<br>Gender binary (0 for male and 1 for female)<br>CentreNo categorical (corresponds to each recruiting centre)<br>irr is incidence rate ratio<br>Treatment effect<br>lincomest _b[1.Medical]
Persistent symptoms	mixed PersistentSymptoms PersistentSymptoms_b i.AgeBand i.Gender i.Medical##i.TimePoint || Centre: || StudyNo:<br>PersistentSymptoms continuous<br>PersistentSymptoms _b is continuous baseline score<br>AgeBand categorical (<35 years; 35-64 years; ≥65 years)<br>Gender binary (0 for male and 1 for female)<br>Medical binary (coded 0 for laparoscopic cholecystectomy and 1 for observation/conservative management)<br>TimePoint is a categorical variable corresponds to the follow-up time points (coded 3 = 3 months, 9 = 9 months, 12 = 12 months, 18 = 18 months and 24 = 24 months)<br>CentreNo categorical (corresponds to each recruiting centre)<br>StudyNo is a unique participant identifier<br><br>Treatment effect for 3 months<br>lincomest _b[1.Medical]<br><br>Treatment effect for 9 months<br>lincomest _b[1.Medical] + _b[1.Medical#9.TimePoint]<br><br>Treatment effect for 12 months<br>lincomest _b[1.Medical] + _b[1.Medical#12.TimePoint]<br><br>Treatment effect for 18 months<br>lincomest _b[1.Medical] + _b[1.Medical#18.TimePoint]<br><br>Treatment effect for 24 months<br>lincomest _b[1.Medical] + _b[1.Medical#24.TimePoint]
AUC up to 24 months post-randomisation for SF-36 bodily pain	mixed auc_24_months i.Medical i.AgeBand i.Gender ||CentreNo:

	auc_24_months continuous (area under the curve from randomisation up to 18 months)<br>Medical binary (coded 0 for laparoscpic cholecystectomy and 1 for observation/conservative management)<br>AgeBand categorical (<35 years; 35-64 years; ≥65 years)<br>Gender binary (0 for male and 1 for female)<br>CentreNo categorical (corresponds to each recruiting centre)<br>Treatment effect<br>lincomest _b[1.Medical]
**Subgroup analysis**	
Gender (male, female)	mixed auc_18_months i.Medical ##i.Gender i.AgeBand ||CentreNo:<br>auc_18_months continuous (area under the curve from randomisation up to 18 months)<br>Medical binary (coded 0 for laparoscopic cholecystectomy and 1 for observation/conservative management)<br>AgeBand categorical (<35 years; 35-64 years; ≥65 years)<br>Gender binary (0 for male and 1 for female)<br>CentreNo categorical (corresponds to each recruiting centre)<br><br>Treatment effect for male<br>lincomest _b[1.Medical]<br><br>Treatment effect for female<br>lincomest _b[1. Medical] + _b[1. Medical#1.Gender]<br><br>Interaction between groups<br>lincomest _b[1.Medical#1.Gender]
Age (<35; 35-64; ≥ 65)	mixed auc_18_months i.Medical ##i.AgeBand i.Gender ||CentreNo:<br><br>auc_18_months continuous (area under the curve from randomisation up to 18 months)<br>Medical binary (coded 0 for laparoscopic cholecystectomy and 1 for observation/conservative management)<br>AgeBand categorical (<35 years; 35-64 years; ≥65 years)<br>Gender binary (0 for male and 1 for female)<br>CentreNo categorical (corresponds to each recruiting centre)<br>Treatment effect for age <35<br>lincomest _b[1.Medical]<br><br>Treatment effect for age 35-64<br>lincomest _b[1.Medical] + _b[1. Medical#1.AgeBand]

	Treatment effect for age ≥ 65<br>lincomest _b[1.Medical] + _b[1. Medical#2.AgeBand]<br><br>Interaction between age <35 and age between 35-64<br>lincomest _b[1.Medical#1.AgeBand]<br><br>Interaction between age <35 and age between ≥ 65<br>lincomest _b[1.Medical#2.AgeBand]
Ethnic groupings (white; mixed/multiple ethnic groups; Asian/Asian British; Black/African/Caribbean/Black British; Other)	mixed auc_18_months i.Medical ##i.Ethnicity i.Gender i.AgeBand ||CentreNo:<br><br>auc_18_months continuous (area under the curve from randomisation up to 18 months)<br>Medical binary (coded 0 for laparoscopic cholecystectomy and 1 for observation/conservative management)<br>Ethnicity categorical (code 0 for white, 1 mixed/multiple ethnic groups, 2 for Asian/Asian British, 3 for black/African/Caribbean/Black British and 4 for other)<br>AgeBand categorical (<35 years; 35-64 years; ≥65 years)<br>Gender binary (0 for male and 1 for female)<br>CentreNo categorical (corresponds to each recruiting centre)<br>Treatment effect for white<br>lincomest _b[1.Medical]<br><br>Treatment effect for mixed/multiple ethnic groups<br>lincomest _b[1.Medical] + _b[1.Medical#1.Ethnicity]<br><br>Treatment effect for Asian/Asian British<br>lincomest _b[1.Medical] + _b[1.Medical#2.Ethnicity]<br><br>Treatment effect for Black/African/Caribbean/Black British<br>lincomest _b[1.Medical] + _b[1.Medical#3.Ethnicity]<br><br>Treatment effect for Other<br>lincomest _b[1.Medical] + _b[1.Medical#4.Ethnicity]<br><br>Interaction between white and mixed/multiple ethnic groups lincomest _b[1.Medical#1.Ethnicity]<br><br>Interaction between white and Asian/Asian British<br>lincomest _b[1.Medical#2.Ethnicity]

	Interaction between white and Black/African/Caribbean/Black British lincomest _b[1.Medical#3.Ethnicity]<br><br>Interaction between white and other lincomest _b[1.Medical#4.Ethnicity]

Appendix 2

Table S1. Reasons for preference

Reason	N (%)
**Preference**	**N=910**
Patient preferred laparoscopic cholecystectomy	538 (59.1)
Patient preferred to have observation/conservative management	167 (18.4)
Did not want to be randomised	91 (10.0)
Surgeon had preference for laparoscopic cholecystectomy	29 (3.2)
Other reason	24 (2.6)
Preference reason unknown	48 (5.3)
Surgeon had preference for observation/conservative management	13 (1.4)
Age of participants with a preference – mean (SD); n	47.9 (15.7); 902

SD standard deviation

Table S2. Surgery details up to 18 months

**Surgical details**	**Observation/conservative management N=217**	**Laparoscopic cholecystectomy N=217**
Received Surgery		
Yes	54 (24·9)	146 (67·3)
No	163 (75·1)	71 (32·7)
	**Received Surgery N=54**	**Received Surgery N=146**
Time to surgery (months) - median (25th and 75th centile); n	8·1 [4·0, 10·6]; 53	4·5 [2·7, 6·9]; 146
Time between surgery and 18 months' follow-up (months) - median (25th and 75th centile); n	9·9 [7·4, 13·0]; 53	13·5 [11·1, 15·5]; 146
Length of hospital stay (days) - median (IQR); n	1 [0, 1]; 51	0 [0, 1]; 143
Operation time (minutes) - median (25th and 75th centile); n	65 [50, 97]; 47	62 [50, 86]; 132
Elective surgery		
Yes	46 (85·2)	142 (97·3)
No	6 (11·1)	2 (1·4)
Missing	2 (3·7)	2 (1·4)
Procedure Type		
Laparoscopic	51 (94·4)	142 (97·3)
Open	1 (1·9)	1 (0·7)
Laparoscopic converted to open	1 (1·9)	1 (0·7)
Missing	1 (1·9)	2 (1·4)
Grade of operating surgeon		
Consultant	31 (57·4)	95 (65·1)
Consultant supervised by another consultant	4 (7·4)	7 (4·8)
Registrar	2 (3·7)	6 (4·1)
Registrar supervised by a consultant	7 (13·0)	19 (13·0)
Specialty (specialty and associate specialist grade) supervised by a consultant	2 (3·7)	-
Senior House Officer supervised by a consultant	1 (1·9)	2 (1·4)
Specialist trainee	-	2 (1·4)
Specialist trainee supervised by a consultant	1 (1·9)	4 (2·7)
Other	-	2 (1·4)
Other supervised by a consultant	2 (3·7)	5 (3·4)
Unknown operating surgeon but supervised by a consultant	-	1 (0·7)
Missing	4 (7·4)	3 (2·1)

Prophylactic antibiotic used in the operation		
Yes	31 (57·4)	77 (52·7)
No	18 (33·3)	61 (41·8)
Missing	5 (9·3)	8 (5·5)
Difficulty of surgery[1]		
Straightforward	28 (51·9)	92 (63·0)
Mildly difficult	5 (9·3)	12 (8·2)
Moderately difficult	3 (5·6)	15 (10·3)
Extremely difficult	3 (5·6)	-
Missing	15 (27·8)	27 (18·5)
Admitted to ICU or HDU		
No	48 (88·9)	138 (94·5)
ICU	-	2 (1·4)
HDU	1 (1·9)	-
Missing	5 (9·3)	6 (4·1)
Time in ICU (hours) - median (25th and 75th centile); n	-	30 [24, 36]; 2
Time if HDU (hours) - value; n	47 [47, 47]; 1	-
Required additional pain relief	12 (22·2)	30 (20·5)
Histopathology		
Normal gallbladder		
Yes	3 (5·6)	7 (4·8)
No	47 (87·0)	136 (93·2)
Missing	4 (7·4)	3 (2·0)
Cholecystitis for abnormal gallbladder		
No	4 (8·5)	7 (5·2)
Acute	4 (8·4)	5 (3·7)
Chronic	38 (80·9)	124 (91·2)
Missing	1 (2·1)	-
Incidental biliary cancer		
No	50 (92·6)	142 (97·3)
Missing	4 (7·4)	4 (2·7)

[1] Completed by the operating surgeon.
HDU High Dependency Unit; ICU Intensive Care Unit;
Values are number (percent) unless otherwise stated

Table S3. Baseline characteristics by those that had and did not have surgery for those randomised to laparoscopic cholecystectomy

	Laparoscopic cholecystectomy N=217	
	**Surgery N=153**	**No surgery N=64**
Age (years) - mean (SD); n	50.5 (15.3); 153	50.7 (15.5); 64
Sex – n (%)		
Male	30 (19.6)	17 (26.6)
Female	123 (80.4)	47 (73.4)
Ethnicity – n (%)		
White	132 (86.3)	56 (87.5)
Mixed/ Multiple ethnic groups	1 (0.7)	-
Asian/ Asian British	12 (7.8)	3 (4.7)
Black/ African/ Caribbean/ Black British	4 (2.6)	1 (1.6)
Arab	1 (0.7)	1 (1.6)
Other	3 (2.0)	3 (4.7)
Missing	-	-
BMI (kg/m^2) - mean (SD); n	30.8 (6.4); 153	33.1 (8.3); 64
Diagnosed with diabetes – n (%)		
No	144 (94.1)	59 (92.2)
Type 1	2 (1.3)	-
Type 2	7 (4.6)	5 (7.8)
Gallbladder wall[1] – n (%)		
Normal	88 (57.5)	32 (50.0)
Thick	19 (12.4)	11 (17.2)
Not recorded	46 (30.1)	21 (32.8)
Thickness of gallbladder wall if thick[1] (mm) - mean (SD); n	6.5 (3.9); 11	4.5 (0.3); 4
Hypertension – n (%)		
No	129 (84.3)	53 (82.8)
Yes	24 (15.7)	11 (17.2)
Missing	-	-
SF-36 norm-based scores - mean (SD);n		
Bodily pain	42.94 (11.12); 153	44.34 (11.12); 63
Physical functioning	47.98 (10.13); 153	45.65 (12.59); 63
Role physical	47.22 (10.83); 153	44.45 (12.56); 63
General health	44.36 (9.94); 153	40.89 (11.06); 63
Vitality	44.97 (10.79); 153	43.96 (11.24); 63
Social functioning	44.48 (11.94); 153	42.48 (13.72); 63
Role emotional	45.06 (12.88); 153	43.97 (14.44); 63
Mental health	46.61 (10.64); 153	44.69 (11.99); 63
PCS	46.11 (9.29); 153	44.35 (10.45); 63
MCS	45.13 (11.79); 153	43.74 (12.82); 63
Otago gallstones CSQ - mean (SD); n	35.73 (19.15); 151	34.58 (24.02); 60
Persistent symptoms score[2] - mean (SD); n	45.96 (22.23); 153	41.33 (24.02); 62

[1] Confirmed by transabdominal ultrasonography or another imaging technique. [2] Derived from two CSQ domains, pain and dyspepsia.

BMI Body Mass Index; CSQ Condition-Specific Questionnaire for gallstones; MCS Mental Component Summary; PCS Physical Component Summary; SD Standard Deviation. For SF-36 norm-based scores, a higher score indicates better quality of life. For Otago gallstones CSQ, higher score indicating higher symptom burden and therefore poorer quality of life ranging from 0-100.

Table S4. Primary outcome – area under the curve SF-36 norm based bodily pain score over 18 months - sensitivity and complete case analysis

Primary outcome	Observation/conservative management N=217	Laparoscopic cholecystectomy N=217
**Sensitivity analysis**[1]		
Baseline	44·5 (11·7); 199	43·4 (11·2); 201
3 months	44·6 (11·5); 176	42·6 (11·0); 174
9 months	46·6 (11·4); 144	47·9 (12·7); 160
12 months	48·6 (11·6); 156	49·0 (11·4); 149
18 months	49·4 (11·7); 167	50·4 (11·6); 161
AUC over 18 months	46·7 (8·8); 200	46·8 (8·7); 201
MD, 95% CI; p-value	-0·0	(-1·8, 1·7); 0·97
**Complete case analysis**[2]		
Baseline	45·0 (11·6); 167	44·2 (11·2); 161
3 months	45·5 (11·5); 147	42·6 (10·4); 145
9 months	46·7 (11·3); 132	47·9 (12·8); 133
12 months	48·7 (11·5); 147	49·1 (11·2); 130
18 months	49·4 (11·7); 167	50·4 (11·6); 161
AUC over 18 months	47·3 (8·8); 167	47·0 (8·4); 161
MD, 95% CI; p-value	0·4	(-1·5, 2·2); 0·69

[1] all participants that had at least one time point up to 18 months. [2]participants that have at least 18 months score. AUC Area Under the Curve; CI Confidence Interval; ITT Intention-to-treat. MD Mean difference. Values are mean (standard deviation); n unless otherwise stated.

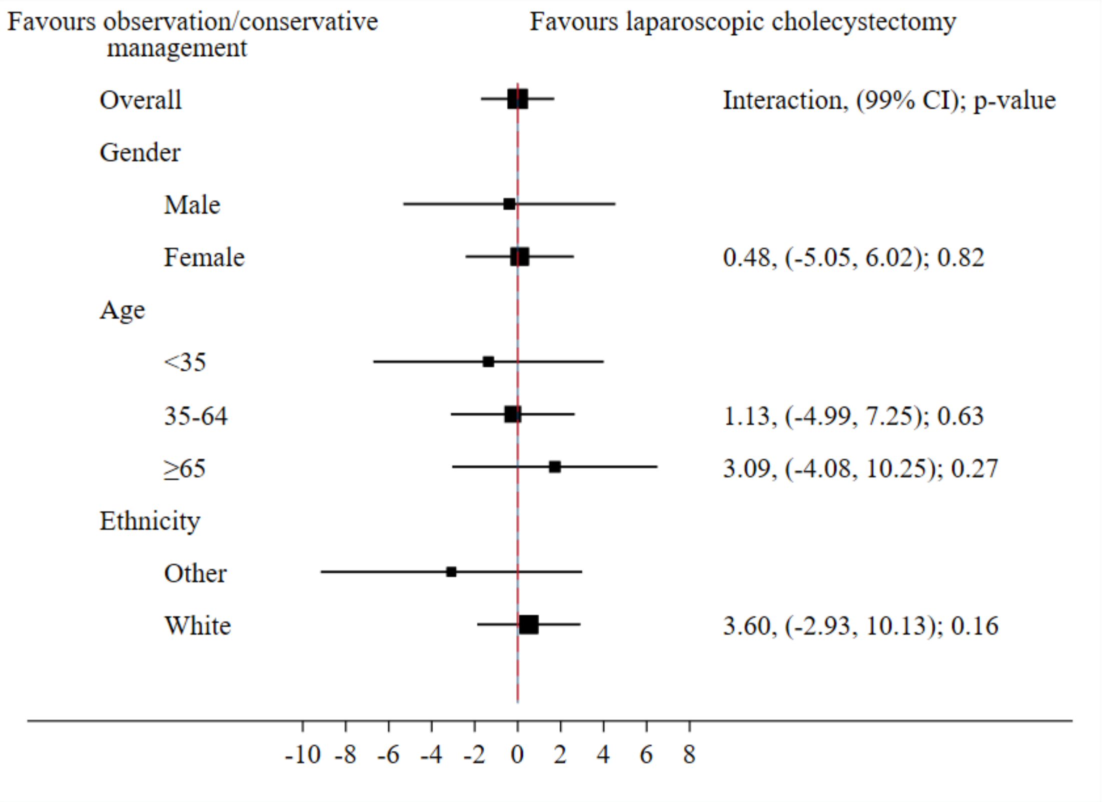

Figure S1: Subgroups for observation/conservative management versus laparoscopic cholecystectomy up to 18 months for SF-36 bodily pain primary analysis

Table S5. Primary outcome – area under the curve SF-36 norm based bodily pain score over 18 months compliance analysis

**Primary Outcome**	**Observation/conservative management N=217**		**Laparoscopic cholecystectomy N=217**	
	**Complied[1]**	**Not-complied[2]**	**Complied[3]**	**Not-complied[4]**
**Primary analysis**				
Baseline	46·3 (11·1); 142	40·3 (11·8); 60	43·1 (11·1); 147	44·1 (11·7); 58
3 months	46·4 (10·8); 124	40·1 (12·0); 52	42·0 (11·0); 126	44·2 (10·7); 48
9 months	46·0 (10·8); 103	48·1 (12·9); 41	48·7 (12·5); 119	45·6 (13·1); 41
12 months	47·8 (11·0); 118	50·8 (13·3); 38	50·0 (10·6); 112	45·9 (13·1); 37
18 months	48·9 (11·6); 121	50·6 (11·8); 46	51·5 (11·1); 119	47·3 (12·3); 42
24 months	46·8 (11·9); 102	51·9 (11·7); 33	49·9 (11·5); 100	47·1 (14·1); 38
AUC over 18 months	47·2 (8·6); 143	45·8 (9·0); 60	47·2 (8·2); 147	45·6 (9·8); 58
MD, 95% CI; p-value			-0·0	(-5·1, 5·1); 0·997

[1] received observation/conservative management.[2] received laparoscopic cholecystectomy.[3] received laparoscopic cholecystectomy.[4] received observation/conservative management.
AUC Area Under the Curve; CI Confidence Interval; ITT Intention to treat; MD Mean difference.
Values are mean (standard deviation); n unless otherwise stated. Compliance was defined as participants who received their allocated treatment within 24 months (apart from the laparoscopic cholecystectomy group if surgery was an emergency).

Table S6. Secondary outcomes – quality of life

	Observation/conservative management N=217	Laparoscopic cholecystectomy N=217	MD	95% CI
**SF-36**				
**Physical Functioning**				
Baseline	48·2 (10·6); 214	47·3 (10·9); 216		
3 months	47·3 (11·0); 161	46·6 (11·1); 155	0·5	(-1·3, 2·3)
9 months	46·5 (11·6); 131	47·4 (11·6); 143	-1·4	(-3·3, 0·4)
12 months	47·7 (10·6); 127	48·2 (11·1); 125	-0·4	(-2·3, 1·5)
**Role Physical**				
Baseline	47·7 (10·3); 215	46·4 (11·4); 216		
3 months	46·8 (10·9); 161	44·8 (11·5); 152	2·1	(0·07, 4·14)
9 months	46·1 (11·5); 130	46·1 (12·5); 138	-0·5	(-2·63, 1·70)
12 months	46·7 (11·8); 126	48·8 (10·9); 121	-1·6	(-3·80, 0·67)
**General Health**				
Baseline	45·0 (9·3); 213	43·3 (10·4); 216		
3 months	43·1 (10·8); 160	42·3 (10·3); 155	0·0	(-1·7, 1·8)
9 months	43·8 (10·7); 130	44·6 (10·8); 139	-1·5	(-3·3, 0·4)
12 months	44·5 (10·6); 127	45·2 (10·6); 125	-1·7	(-3·5, 0·2)
**Vitality**				
Baseline	46·7 (10·0); 213	44·7 (10·9); 216		
3 months	44·8 (10·8); 160	44·2 (10·5); 156	-0·6	(-2·5, 1·3)
9 months	44·9 (10·9); 130	46·3 (11·3); 139	-2·9	(-5·0, -0·9)
12 months	45·9 (11·5); 127	46·7 (11·2); 125	-2·1	(-4·2, 0·0)
**Social Functioning**				
Baseline	45·6 (11·7); 213	43·9 (12·5); 216		
3 months	43·9 (11·9); 161	42·5 (12·4); 155	0·8	(-1·4, 3·1)
9 months	44·1 (11·6); 129	46·1 (12·4); 140	-2·4	(-4·8, 0·0)
12 months	44·5 (13·0); 126	46·2 (12·2); 124	-1·5	(-4·0, 1·0)
**Role Emotional**				
Baseline	45·9 (12·4); 215	44·7 (13·3); 216		
3 months	44·5 (12·6); 160	42·7 (12·8); 152	1·8	(-0·5, 4·1)
9 months	44·2 (12·7); 129	44·6 (13·6); 138	-1·5	(-4·0, 1·0)
12 months	44·7 (12·8); 124	46·3 (12·4); 121	-1·7	(-4·2, 0·9)
**Mental Health**				
Baseline	47·7 (10·4); 213	46·1 (11·1); 216		
3 months	44·9 (11·4); 160	45·1 (11·4); 156	-0·7	(-2·8, 1·3)
9 months	45·2 (11·4); 130	47·2 (11·2); 139	-2·8	(-4·9, -0·6)
12 months	46·4 (12·4); 127	47·1 (11·6); 125	-1·6	(-3·8, 0·6)
**Physical component summary - PCS**				
Baseline	46·7 (9·3); 213	45·6 (9·7); 216		
3 months	46·3 (10·1); 157	44·8 (10·2); 150	1·4	(-0·4, 3·2)

9 months	46·4 (10·4); 127	46·9 (11·7); 136	-0·8	(-2·7, 1·1)
12 months	47·4 (10·8); 123	48·4 (10·5); 119	-0·8	(-2·7, 1·2)
**Mental component summary – MCS**				
Baseline	46·4 (11·5); 213	44·7 (12·1); 216		
3 months	43·9 (12·3); 157	43·2 (12·4); 150	-0·1	(-2·3, 2·1)
9 months	44·2 (12·4); 127	46·0 (11·7); 136	-2·8	(-5·1, -0·5)
12 months	45·1 (13·3); 123	46·1 (11·9); 119	-1·8	(-4·2, 0·6)
**CSQ Total**				
Baseline	33·2 (19·9); 210	35·4 (20·6); 211		
3 months	29·5 (23·4); 148	30·9 (22·6); 147	-0·8	(-4·9, 3·4)
9 months	26·6 (22·5); 122	23·6 (22·4); 132	4·4	(-0·0, 8·8)
12 months	21·7 (22·1); 119	18·4 (19·4); 120	4·7	(0·2, 9·2)
**Persistent symptoms score**[1]				
Baseline	43·0 (20·9); 213	44·6 (22·8); 215		
3 months	32·9 (26·6); 156	34·6 (24·7); 153	-1·4	(-6·4, 3·5)
9 months	31·1 (26·5); 128	26·7 (26·1); 139	5·5	(0·3, 10·8)
12 months	23·4 (24·2); 125	20·2 (23·1); 121	4·6	(-0·9, 10·0)

[1] derived from two CSQ domains, pain and dyspepsia.
CI Confidence Interval; CSQ Condition Specific Questionnaire; MD Mean Difference; MSC the Mental Component Summary; PCS Physical Component Summary.
Values are mean (standard deviation), n; For SF-36 norm-based scores, a higher score indicates better quality of life; For Otago gallstones CSQ, higher score indicating higher symptom burden and therefore poorer quality of life.

Table S7. Secondary outcome – complications up to 24 months

	Observation/conservative management N=217	Laparoscopic cholecystectomy N=217
Number of participants	32 (14·7)	46 (21·2)
RR (95% CI); p-value	0·69	95% CI (0·44, 1·09); p-value 0·11
Number of complications		
1	18	32
2	8	5
3	4	8
4	2	1
**Pre-surgery complications**		
Number of participants	25 (11·5)	11 (5·1)
Number of complications		
1	20	9
2	4	
3	1	1
4		1
**Details of pre-surgery complications**		
Cholecystitis	14	8
Biliary colic	8	2
Pancreatitis	2	3
Choledocholithiasis	2	-
Cholecystitis and jaundice	1	-
Choledocholithiasis and pancreatitis	1	-
**Clinical secondary outcome**	**Observation/conservative management N=217**	**Laparoscopic cholecystectomy N=217**
Details of pre-surgery complications continued		
Cholecystitis, choledocholithiasis and jaundice	-	1
Cholecystitis and pancreatitis	1	-
Bouveret's Syndrome[1]	1	-
Cholecystitis, choledocholithiasis and pancreatitis	-	1
Jaundice	-	1
Right upper quadrant pain	1	-
**Intra-operative complications**		
Number of participants	9 (4·1)	24 (11·1)
Number of complications		
1	8	23
2	1	1
**Details of intra-operative complications**		
Bile/stone spillage from gall bladder	6	16

Injury to abdominal viscera (including liver tear or laceration)	1	5
Bleeding >500ml	1	2
Bile leak from the bile duct, hepatic duct, or ducts at base of liver	1	1
Injury to bile duct	1	-
Ruptured empyema	-	1
**Post-operative complications**		
Number of participants	7 (3·2)	16 (7·4)
Number of complications		
1	5	10
2	1	4
3	1	3
**Details of post-operative complications**		
Bleeding >500ml	1	2
Bile leak that required no treatment	2	3
Bowel obstruction requiring no treatment	1	4
Bowel obstruction requiring surgery	-	1
Wound infection	2	2
Intraperitoneal - collection/abscess requiring no treatment	1	4
Intraperitoneal - collection/abscess requiring pre-cutaneous drainage	1	-
Vomiting	-	3
Dizziness and hypotension	1	-
Haematoma	-	1
Missed stone in the bile duct	-	1
Renal failure	-	1
Residual gallbladder inflamed	1	-
Wound dehiscence	-	1
**Post-surgery complications within 30 days of discharge**		
Number of participants	2 (0·9)	3 (1·4)
Number of complications		
1	2	3
**Details of post-surgery complications within 30 days of discharge**		
Cholangitis	-	1
Surgical site infection	1	1
Bile leak	-	1
Post-cholecystectomy syndrome[2]	1	-
**Post-surgery complications after 30 days of discharge**		
Number of participants	1 (0·5)	1 (0·5)

**Details of post-surgery complications after 30 days of discharge**		
Right upper quadrant pain	-	1
Incisional hernia	1	-
**Death - Cardiovascular event**		
Number of participants	-	1 (0·5)

[1] Bouveret's syndrome occurs when a gallstone enters the small bowel via a bilioenteric fistula and is impacted in the duodenum or stomach, causing gastric outlet obstruction.
[2] Persistence of same symptoms reported by the patient post-surgery.
CI Confidence Interval; RR Relative Risk; Values are numbers (percent) or numbers.

Table S8. Secondary outcome – further treatment up to 24 months

	**Observation/conservative management N=202[1]**	**Laparoscopic cholecystectomy N=203[1]**
Number of participants with at least one further treatment	10 (5·0)	16 (7·9)
RR (95% CI); p-value	0·62	95% CI (0·28, 1·38); p-value 0·24
Number of treatments		
1	8	10
2	2	4
3	-	1
4	-	1
**Details of the further treatment[2]**		
Pain relief	3	12
Antibiotics	2	5
ERCP	3	4
Anti-sickness	1	-
Bloating	1	-
Urinary catheter for retention	-	2
Bowel	-	1
Colostomy	1	-
Blood transfusion	-	1
Laparotomy washout and haemostasis	-	1
Fluids	-	1
Pancreatitis management	-	1
Unknown	1	-

[1] number followed up. [2] corresponding to the number of events. CI Confidence Interval; ERCP endoscopic retrograde cholangiopancreatography; RR Relative Risk.
Values are numbers (percent) or numbers.
